# Survival differences and associated molecular signatures of *DNMT3A*-mutant acute myeloid leukemia patients

**DOI:** 10.1038/s41598-020-69691-8

**Published:** 2020-07-29

**Authors:** Chris Lauber, Nádia Correia, Andreas Trumpp, Michael A. Rieger, Anna Dolnik, Lars Bullinger, Ingo Roeder, Michael Seifert

**Affiliations:** 1 Institute for Medical Informatics and Biometry (IMB), Carl Gustav Carus Faculty of Medicine, Technische Universität Dresden, Dresden, Germany; 2 Division of Stem Cells and Cancer, German Cancer Research Center (DKFZ), Heidelberg, Germany; 3 Department of Medicine, Hematology/Oncology, Goethe University Hospital Frankfurt, Frankfurt, Germany; 4 Department of Hematology, Oncology and Tumorimmunology, Charité University Medicine Berlin, Campus Virchow Klinikum, Berlin Germany; 5 National Center for Tumor Diseases (NCT), Dresden, Germany

**Keywords:** Data mining, Acute myeloid leukaemia

## Abstract

Acute myeloid leukemia (AML) is a very heterogeneous and highly malignant blood cancer. Mutations of the DNA methyltransferase *DNMT3A* are among the most frequent recurrent genetic lesions in AML. The majority of *DNMT3A*-mutant AML patients shows fast relapse and poor survival, but also patients with long survival or long-term remission have been reported. Underlying molecular signatures and mechanisms that contribute to these survival differences are only poorly understood and have not been studied in detail so far. We applied hierarchical clustering to somatic gene mutation profiles of 51 *DNMT3A*-mutant patients from The Cancer Genome Atlas (TCGA) AML cohort revealing two robust patient subgroups with profound differences in survival. We further determined molecular signatures that distinguish both subgroups. Our results suggest that *FLT3* and/or *NPM1* mutations contribute to survival differences of *DNMT3A*-mutant patients. We observed an upregulation of genes of the p53, VEGF and DNA replication pathway and a downregulation of genes of the PI3K-Akt pathway in short- compared to long-lived patients. We identified that the majority of measured miRNAs was downregulated in the short-lived group and we found differentially expressed microRNAs between both subgroups that have not been reported for AML so far (*miR-153-2*, *miR-3065*, *miR-95*, *miR-6718*) suggesting that miRNAs could be important for prognosis. In addition, we learned gene regulatory networks to predict potential major regulators and found several genes and miRNAs with known roles in AML pathogenesis, but also interesting novel candidates involved in the regulation of hematopoiesis, cell cycle, cell differentiation, and immunity that may contribute to the observed survival differences of both subgroups and could therefore be important for prognosis. Moreover, the characteristic gene mutation and expression signatures that distinguished short- from long-lived patients were also predictive for independent *DNMT3A*-mutant AML patients from other cohorts and could also contribute to further improve the European LeukemiaNet (ELN) prognostic scoring system. Our study represents the first in-depth computational approach to identify molecular factors associated with survival differences of *DNMT3A*-mutant AML patients and could trigger additional studies to develop robust molecular markers for a better stratification of AML patients with *DNMT3A* mutations.

## Introduction

Acute myeloid leukemia (AML) is a highly malignant cancer of myeloid blood cells affecting about one million people globally in 2015^1,2^. It most frequently occurs in older adults and shows a relatively poor five-year survival rate of about 25%, which is worsening with increasing age of a patient at diagnosis^3^. AML is characterized by a rapid growth of abnormal, immature myeloblasts that lost their ability to differentiate, which replace normal cells in the bone marrow and blood. At the level of underlying genetic aberrations, AML is very heterogeneous. Mutations in several genes are required for leukemic transformation affecting multiple steps of the differentiation pathway^4,5^. In addition, different cytogenetic abnormalities of significant prognostic relevance, ranging from translocations (t(8;21), t(15;17)) and inversions (inv(16)) with relatively good prognosis to deletions of whole chromosomes (5, 7) or chromosomal arms (5q) and abnormalities on the q-arm of chromosome 3 (3q) associated with high risk, have been observed in AML patients^6–8^.

The first genome of a cytogenetically normal AML patient was sequenced in 2008^9^. The Cancer Genome Atlas (TCGA) Research Network made enormous efforts to perform whole-genome or exome sequencing, transcriptome and microRNA (miRNA) sequencing, and DNA methylome analysis of a large cohort of adult AML cases in 2013^10^. These and other sequencing-based studies (e.g.^11–14^) enabled the identification of several genetic and genomic alterations acquired during AML pathogenesis. Subtypes of AML are associated with distinctive patterns of altered gene expression (e.g.^15–17^). Likewise, a prognostic and functional role of widespread dysregulation of miRNAs has emerged^18,19^. Regarding somatic mutations, it was found that only about a dozen genes are affected on average in an AML patient, which is considerably less than in most other human cancers^10^. The by far top-ranking recurrently mutated genes in AML are *FLT3*, *NPM1* and *DNMT3A*^10^.

The DNA methlytransferase 3A (*DNMT3A*) forms a gene family of DNA methyltransferases together with *DNMT3B* and *DNMT1*, where the encoded proteins DNMT3A and DNMT3B add methyl groups to unmodified DNA by conversion of cytosine to 5-methylcytosine, while DNMT1 maintains existing DNA methylation after cell division^20^. *DNMT3A* is highly expressed in embryonic stem cells^21,22^. A *DNMT3A* deletion in mouse hematopoietic stem cells has been shown to inhibit differentiation^23^ and a deletion of *DNMT3A* in human hematopoietic stem cells resulted in increased self-renewal and blockage of differentiation^24^. This importance of *DNMT3A* for normal hematopoiesis is in line with its high frequency of somatic mutations in AML, which are found in about 20% of patients^9,25^. It is assumed that *DNMT3A* mutations are acquired months or years before a potential onset of AML from hematopoietic stem cells or multipotent precursor cells leading to a pre-leukemic state that potentially leads to the development of AML^26,27^. In addition, significant associations of *DNMT3A* mutations with IDH1/2 mutations, *FLT3* internal tandem duplications (ITD) and tyrosine kinase domain mutations (TKD), and *NPM1* mutations have been reported^9,28^.

Notably, around two-thirds of the *DNMT3A* mutations affect the R882 codon in the methyltransferase domain of *DNMT3A*^9,25^. Moreover, *DNMT3A* mutations in general or those affecting the R882 residue have been linked to shorter survival rates of patients^9,14,25,29–31^, but there is also an ongoing debate about the prognostic values of R882 and non-R882 *DNMT3A* mutations. This debate is fueled by the fact that, in contrast to generally poor prognosis, some *DNMT3A*-mutant patients show relatively long survival or even go into long-term remission with *DNMT3A* mutations remaining stable^32,33^. Molecular characteristics associated with such prognosis differences of *DNMT3A*-mutant patients have not been intensively studied so far.

Here, we initially analyzed genome-wide somatic mutation profiles of *DNMT3A*-mutant patients from the TCGA AML cohort by hierarchical clustering. Our analysis revealed two patient subgroups with profound differences in overall survival rates. Additional analyses of gene and miRNA expression data in combination with inference of gene regulatory networks enabled us to identify molecular patterns of expression dysregulation as well as gene modules that distinguish both subgroups. The characteristic gene mutation and expression signatures also enabled to separate *DNMT3A*-mutant AML patients of two independent cohorts into a short- and long-lived group. The results of our computational analysis point toward several genetic regulators and cellular processes that are potentially involved in a manifestation of apparent survival differences of AML patients with *DNMT3A* mutations.

**Figure 1:**
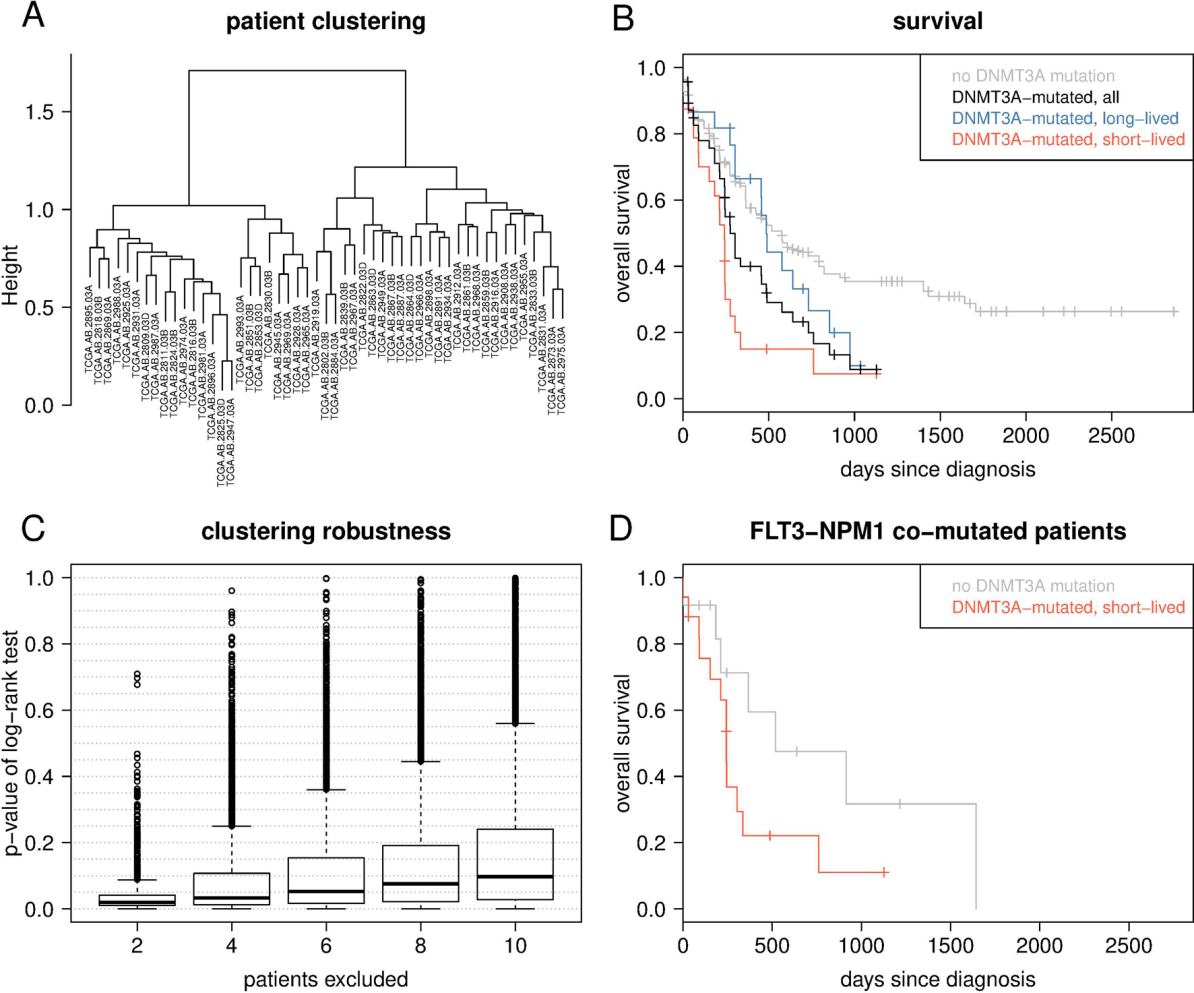
Clustering of *DNMT3A*-mutated AML patients into two subgroups that differ in survival. (**A**) Hierarchical clustering of 51 *DNMT3A*-mutated AML patients; tip labels indicate TCGA identifiers (left subtree: short-lived, right subtree: long-lived). (**B**) Kaplan-Meier survival curves for the patients from (**A**) (black) and the two subgroups (short-lived: red, left subtree in A, survival data available for all 24 patients; long-lived: blue, right subtree in A, survival data available for 23 of 27 patients) as well as for 138 AML patients without a *DNMT3A* mutation (gray). Log-rank tests: P < 0.013 for red vs. blue, P < 0.0001 for red vs. grey, P = 0.345 for blue vs. grey, P = 0.004 for black vs. grey. (**C**) Robustness of clustering the *DNMT3A*-mutated patients into two subgroups that differ in survival, as assessed by randomly excluding patients and performing a hierarchical clustering and subsequent log-rank test on the data subset. Each boxplot shows the distributions of p-values of the log-rank tests for 10,000 data subsets. (**D**) Kaplan-Meier survival curves analyzing the impact of *FLT3* and *NPM1* co-mutations for all 17 affected patients of the short-lived subgroup (red) and all 12 affected patients of the 138 patients without a *DNMT3A*-mutation (grey). Log-rank test: P < 0.094.

**Figure 2:**
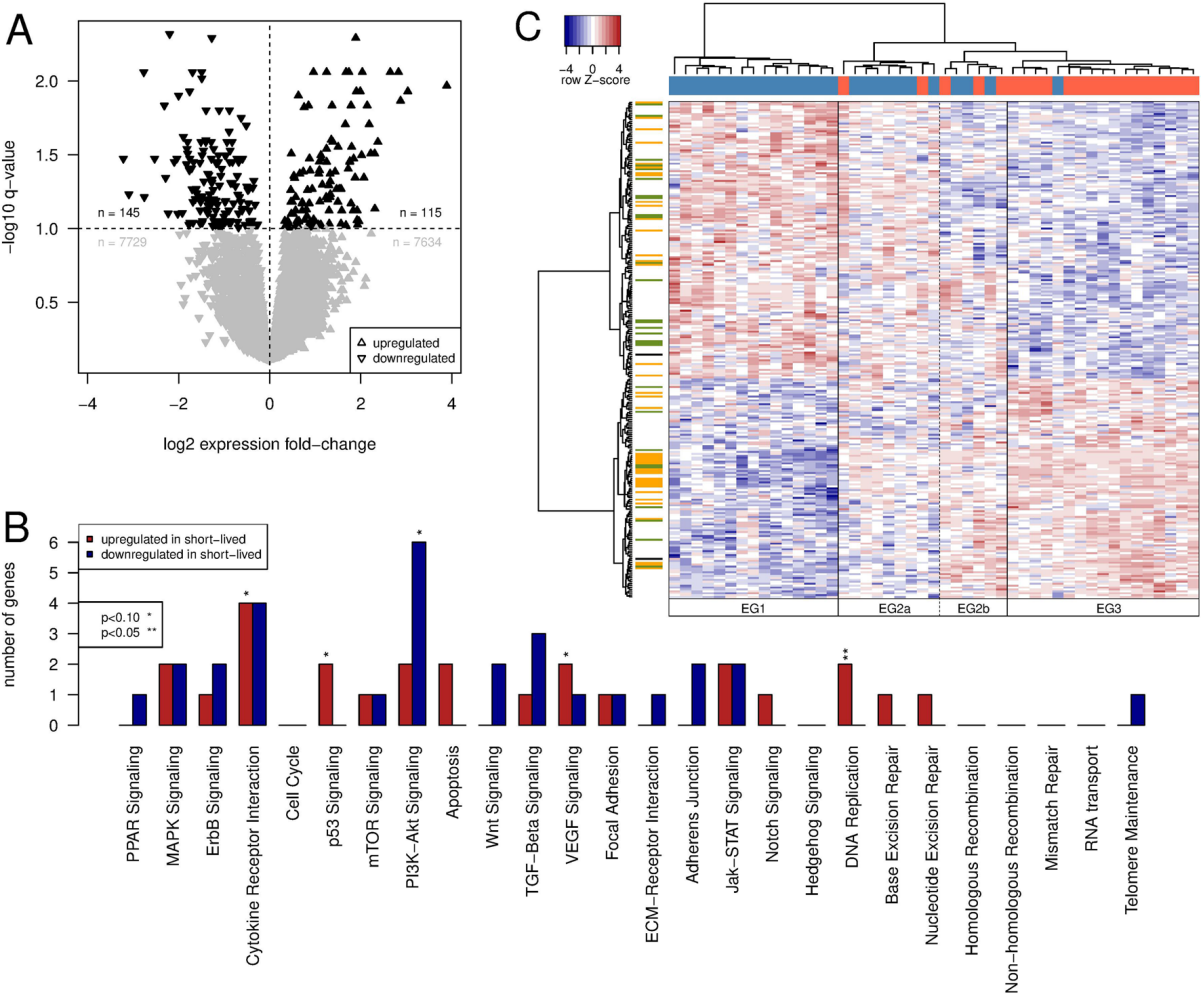
Genes differentially expressed between patient subgroups and enrichment analysis. (**A**) Volcano plot showing the relative expression change of the 15,623 genes between patients from the short-lived and long-lived subgroup. Genes with a significant change in expression (q < 0.1) are in black, others in gray. (**B**) Signaling pathways enriched with genes that are differentially expressed between the short- and the long-lived subgroup; separately shown for genes upregulated (red) and downregulated (blue), respectively, in the short-lived relative to the long-lived subgroup. (**C**) Gene expression heatmap of 260 differentially expressed genes. Rows are Z score-scaled. Column coloring indicates patients from the short-lived (red) and the long-lived (blue) subgroup. Row coloring highlights known transcription factors (yellow), genes involved in signaling pathways (green) and genes showing both of these annotations (black).

## Results

### Two subgroups of *DNMT3A*-mutated AML patients differ in overall survival

Considering the gene mutation data from TCGA for all 197 AML patients, we found that 51 of them had a *DNMT3A* mutation. We observed in total 5 frame-shift, 43 missense, 6 nonsense and 3 splice site *DNMT3A* mutations including 6 patients that had two of these mutations. For 29 (57%) of the patients, the mutation affected the R882 codon at second (n=22) or first (n=7) codon position (Supplementary Table 1). The 51 *DNMT3A*-mutated patients had on average 13.3 mutated genes (min=2, max=24) from 1,890 genes analyzed in total. Hierarchical clustering of the 51 *DNMT3A*-mutated patients based on binary mutational profiles of the 1,890 genes revealed two well-separated subgroups of nearly equal size (24 vs. 27 patients; Fig. 1A). Importantly, the two subgroups of *DNMT3A*-mutant patients showed a significant difference in overall survival (P < 0.013; Fig. 1B, Supplementary Table 2). Compared to 138 AML patients without a *DNMT3A* mutation, only the subgroup with shorter overall survival (short-lived subgroup from here on) showed a statistically significant difference in survival (P < 0.0001), while the other (long-lived subgroup) did not (P < 0.345), although a considerable deviation of its survival curve from that of the non-mutated patients was observed (Fig. 1B). Generally, *DNMT3A*-mutant patients showed significantly shorter survival than patients without a *DNMT3A* mutation (Fig. 1B, P = 0.004). Further, the short-lived subgroup was enriched with patients harboring a R882 *DNMT3A* mutation (n=17, 71%) compared to patients with non-R882 mutations (n=7, 29%), while the long-lived subgroup was composed of 12 patients with R882 (44%) and 15 patients with non-R882 mutations (56%). However, this difference in the proportion of R882 mutations of both subgroups was not significant (Chi-squared test, P = 0.106). We further compared the number of mutated genes and cytogenetic abnormalities between the short- and long-lived subgroup. The median number of mutated genes of short-lived patients was significantly smaller than for long-lived patients (Supplementary Fig. 5; U-Test: P < 0.004; short-lived: 10.5; long-lived: 17). The majority of short- (71%) and long-lived patients (59%) had normal cytogenetic profiles. Interestingly, the long-lived group contained 7 patients (26%) with duplications or rearrangements of chromosome 8 that have not been observed in the short-lived group.

To evaluate the robustness of the grouping of the 51 *DNMT3A*-mutated patients into two subgroups that differ in survival, we repeated the clustering for data subsets obtained by excluding different randomly selected fractions of patients considering 10,000 repetitions of this procedure (see Methods for details). For the vast number of subsets, the difference in patient survival between the subgroups remained significant or stayed close to the level of significance obtained for the full data set, although p-values of the log-rank tests increased with increasing number of excluded patients (Fig. 1C). The latter is not unexpected considering the limited number of *DNMT3A*-mutated AML patients.

For the analysis in which two patients were excluded at random, we observed that few subsets showed exceptionally high p-values of the corresponding log-rank tests. The patients excluded from these subsets exclusively belonged to a set of in total 4 members of the short-lived subgroup (TCGA case identifiers: TCGA-AB-2931-03, TCGA-AB-2824-03, TCGA-AB-2896-03, TCGA-AB-2945-03). Each of these four patients died, and their survival times were, respectively, 0, 30, 214, and 243 days after diagnosis. The four patients showed mutations in 13, 5, 3 and 13 genes, respectively; all four had an *NPM1* and two of them an *FLT3* mutation, while the remaining mutations were found only once among the four patients.

### Frequent *FLT3* and *NPM1* mutations distinguish short- and long-lived *DNMT3A*-mutated patients

In order to understand whether and how patients from the two identified subgroups differ at the molecular level, we first searched for somatic mutations of specific genes that were enriched in one subgroup compared to the other. We found that each patient of the short-lived subgroup had at least one of either *FLT3* (20 of 24 patients) or *NPM1* (21 of 24 patients) mutated, with 17 (71%) of them showing mutations in both of these genes. In sharp contrast, *FLT3* and *NPM1* were mutated in only one and seven patients of the 27 patients from the long-lived subgroup, respectively. We did not find any gene with strong enrichment of mutations in patients from the long-lived subgroup. Instead, we only observed slightly increased numbers of five *IDH2* and four *MT-CYB* mutations in this subgroup. These two genes were not mutated in any of the patients from the short-lived subgroup.

To test whether or not the short survival of patients from the short-lived subgroup is mainly driven by *FLT3*-*NPM1* co-mutations, we separately analyzed a subset of in total 29 AML patients from the TCGA AML cohort, which had these two genes mutated. Seventeen of them also had a *DNMT3A* mutation and showed a considerably shorter survival compared to the remaining 12 patients without a *DNMT3A* mutation (Fig. 1D; Log-rank test, P < 0.094). Although not statistically significant but considering the small sample size, this points towards an effect of *DNMT3A* mutations on survival that is independent of *FLT3* and *NPM1* co-mutations.

Also patients either having a *FLT3* mutation or a *NPM1* mutation in combination with a *DNMT3A* mutation showed shorter overall survival than patients without a *DNMT3A* mutation (Supplementary Fig. 2). Further, the overall survival of patients with *NPM1*-*DNMT3A* co-mutations was very similar to those of patients with *FLT3*-*NPM1*-*DNMT3A* co-mutations. Co-mutations of *DNMT3A* with *FLT3*, *NPM1* or both genes were generally associated with poor survival.

In addition, we determined the specific type of *FLT3* mutation for each patient and analyzed if *FLT3*-ITD and *FLT3*-TKD differ in their impact on survival of *DNMT3A*-mutant AML patients from TCGA (Supplementary Table 1, Supplementary Fig. 6). The 20 *FLT3* mutations in the short-lived subgroup were split into 11 *FLT3*-ITD and 9 *FLT3*-TKD mutations. The one *FLT3* mutation in the long-lived group was a *FLT3*-ITD mutation. There was no significant difference in survival of *DNMT3A*-mutant AML patients distinguished by their type of *FLT3* mutation. Both groups did also not significantly differ in survival in comparison to *DNMT3A*-mutant AML patients without *FLT3* mutations.

We further analyzed the gene mutation profiles within the short- and long-lived group by additionally dividing each corresponding subtree in Fig. 1A into its two major patient subgroups (Supplementary Fig. 3). Both derived short-lived subgroups strongly differed in the number of co-mutations of *DNMT3A* with *FLT3* or *NPM1*. The two derived long-lived subgroups strongly differed in the number of co-mutations of *DNMT3A* with *IDH1* or *IDH2* and also in the number of *NPM1* mutations.

Since *DNMT3A*-R882 mutations were increased in the short-lived group, we analyzed if mutations of *FLT3* or/and *NPM1* are found more frequently in AML patients with *DNMT3A*-R882 mutations compared to patients with other *DNMT3A* mutations. We therefore considered a large independent cohort of AML patients^34^ and found a significant enrichment of *DNMT3A*-R882 and *NPM1* co-mutations and a significant enrichment of concurrent *DNMT3A*-R882, *NPM1*, *FLT3* mutations compared to the corresponding groups of patients with other *DNMT3A* mutations (Supplementary Fig. 4, Fisher’s exact test: P < 0.01), whereas no significant difference in the proportion of *FLT3* mutations was found. We also observed systematic differences considering the percentage of peripheral blood blasts, white blood cell counts, platelet counts, and the hemoglobin level indicating that differentiation capabilities of AML cells with R882 and non-R882 *DNMT3A* mutations may differ at least to some extent (Supplementary Fig. 4).

### A gene expression signature discriminates short- and long-lived *DNMT3A*-mutated patients

Next, we used RNA-Seq gene expression data from TCGA for the 51 *DNMT3A*-mutated patients and conducted a differential gene expression analysis to search for genes that differ in their expression levels between the short- and long-lived subgroup. We identified 260 differentially expressed genes (DEGs) using an FDR-corrected p-value (q-value) cut-off of 0.1 (Fig. 2A, Supplementary Table 3).

When grouping the 260 DEGs into different functional categories (transcription factors, oncogenes, tumor suppressor genes, kinases, phosphatases, signaling and metabolic pathway genes, etc.^35^), we only found a significant enrichment for known cancer-relevant signaling pathway genes. This included four genes involved in cytokine receptor interactions (*CCL23, FAS, KITLG, TSLP*) that were upregulated in the short-lived relative to the long-lived subgroup, two genes of the p53 signaling pathway (*FAS, TP53I3*) that were also upregulated, six genes involved in PI3K-Akt signaling (*EFNA1, FGF9, GNG11, GNG2, GNG7, ITGA6*) that were downregulated, two genes of the VEGF signaling pathway (*PIK3CB, PLA2G4A*) that were upregulated, and two genes involved in DNA replication (*POLE4, RNASEH2C*) that were also found to be upregulated in the short-lived subgroup (Fig. 2B).

**Table 1:** Assignment of short- and long-lived patients to our revealed gene expression groups in combination with meta-information about cytogenetic abnormality types and FAB types of the *DNMT3A*-mutant AML patients from TCGA.

	Expression group			
	EG1	EG2a	EG2b	EG3
**Group composition**				
Short-lived patients	0	2	3	16
Long-lived patients	15	7	3	1
**Cytogenetic abnormality types**				
n.a.	1	1	0	2
8+	3	1	0	0
7q-	1	1	0	0
Complex	1	0	0	1
Complex 5p-	1	0	0	0
Normal	7	6	4	14
Normal 8+	1	0	1	0
Normal 7q-	0	0	1	0
**FAB types**				
n.a.	0	1	0	0
M0	2	0	0	0
M1	5	3	0	3
M2	4	3	0	3
M3	0	0	0	1
M4	3	2	1	6
M5	0	0	5	4
M7	1	0	0	0

See also Supplementary Fig. 1 for an overview of the number of mutated genes per subgroup.

**Table 2:** Non-HOX network modules and potential major regulators.

Regulator gene	GeneCards annotation summary
**Network module 1**	
*SLC4A1*	Anion exchanger; role in O2/CO3 exchange in erythrocytes
*HBM*	Hemoglobin subunit Mu; iron ion and oxygen binding
*RHD*	Rh blood group D antigen; ammonium transmembrane transporter activity
*GYPA*	erythrocyte membrane protein; MN blood group receptor; hematopoietic stem cell differentiation; associated with Anemia, Autoimmune Hemolytic
*CA1*	Carbonate dehydratase and hydro-lyase activity; highest concentration in erythrocytes; nitrogen metabolism
**Network module 2**	
*ZAP70*	T cell receptor associated kinase; T cell development; lymphocyte activation
*CD3D*	Part of T cell receptor/CD3 complex; associated with immunodeficiencies
*EVL*	Enhances actin nucleation and polymerization; actin and profilin binding
*IFITM1*	Interferone-induced transmembrane protein; antiviral activity; cell adhesion and control of cell growth and migration; regulates osteoblast differentiation
**Network module 3**	
*TNFRSF17*	TNF receptor of major B lymphocytes; autoimmune response; transduces signals for cell survival and proliferation
*IGKV4-1*	V segment of variable domain of immunoglobulin light chain
*IGKC*	Constant region of immunoglobulin heavy chains
**Network module 4**	
*GYPC*	Erythrocyte membrane protein; Gerbich blood group; response to elevated platelet cytosolic Ca2+; regulation of mechanical cell stability
*MREG*	Melanoregulin; incorporation of pigments into hair; membrane fusing
**Network module 5**	
*SORL1*	Transmembrane signaling receptor activity; low-density lipoprotein binding
*C1QTNF4*	Pro-inflammatory cytokine; activation of NF-kappa-B; IL6 up-regulation
**Network module 6**	
*RPS3*	Ribosomal protein; mRNA activation
*RPS19*	Ribosomal protein; mRNA activation; associated with anemia

**Table 3:** Network miRNAs and potentially directly or indirectly regulated protein-coding genes. The logFC-column quantifies the expression level of the miRNA within the short-lived subgroup relative to the long-lived subgroup.

miRNA	logFC	Connected gene	GeneCards annotation summary
*hsa-let-7b*	1.01	*PAPD7*	Poly(A) RNA polymerase; oncogenic MAPK signaling; DNA repair; sister chromatin adhesion
*hsa-mir-10a*	2.97	*PBX3*	Astrocytoma association; misregulation in cancer; transcription factor activity
*hsa-mir-10a*	2.97	*HOXB3*	Transcription factor in development, host gene of *hsa-mir-10a*
*hsa-mir-128-1*	− 0.54	*ARPP21*	cAMP-regulated phosphoprotein; nucleic acid and calmodulin binding; enriched expression in CNS
*hsa-mir-130a*	− 1.88	*FAM69B*	Cysteine-rich type II transmembrane protein of unknown function
*hsa-mir-150*	− 0.81	*LEF1*	T cell receptor binding; Wnt signaling, cancer association; transcription factor activity
*hsa-mir-196b*	1.16	*HOXA7*	Transcription factor in development
*hsa-mir-486*	− 0.93	*LBH*	transcriptional activator in mitogen-activated protein kinase signaling pathway
*hsa-mir-628*	− 0.60	*BEND2*	participation in protein and DNA interactions during chromatin restructuring or transcription
*hsa-mir-6718*	2.61	*LRMDA*	Leucin-rich; melanocyte differentiation

Since we compared expression profiles of two relatively large groups, individual genes can also vary in their expression within a group while still being differentially expressed between both groups. This can result in additional subgroups that are masked by the global differential expression analysis. We therefore performed a hierarchical clustering of the patients based on expression profiles of the 260 DEGs, which resulted in four expression groups (EGs, Supplementary Table 2) of patients with characteristic large-scale expression differences for the 260 genes (Fig. 2C). EG1 exclusively contained 15 patients from the long-lived subgroup, while EG3 included 16 short-lived and a single long-lived patient (Fig. 2C, Table 1). These two groups with evident differences in gene expression thus strongly resemble the long- and short-lived subgroup clustering based on the somatic mutation data. The other two groups of patients (EG2a and EG2b) represented a mixture of in total five patients from the short-lived and ten patients from the long-lived subgroup with intermediate expression levels for most of the 260 DEGs (Fig. 2C, Table 1).

When inspecting additional meta-information from TCGA for the patients of the different expression groups, we observed no systematic differences regarding cytogenetic abnormality types. Instead, there was a notable tendency that patients of EG2b and EG3, the two groups with a high or very high fraction of short-lived patients, were more frequently classified to have FAB type M4 (acute myelomocytic leukemia) or M5 (acute monoblastic leukemia or acute monocytic leukemia) (Table 1). The FAB types M4 and M5 have previously been associated with a high mutational burden at diagnosis^36^. This was not confirmed for our cohort, where the median number of mutated genes for patients within EG2b and EG3 was significantly smaller than for patients within EG1 and EG2a (U-Test: P < 0.002; EG2b and EG3: 11; EG1 and EG2a: 17; Supplementary Fig. 1).

**Figure 3:**
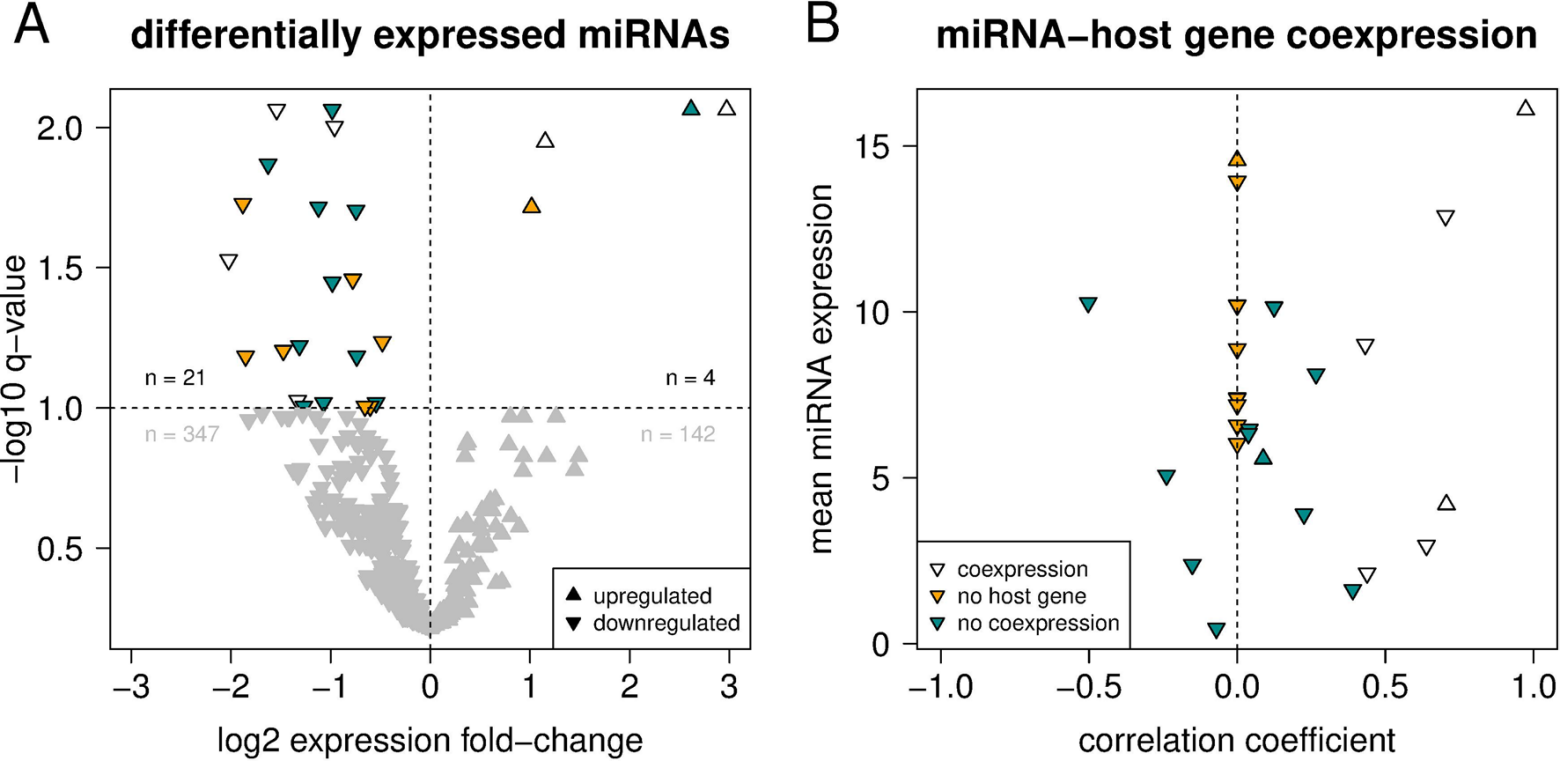
Differentially expressed miRNAs and co-expression of miRNAs and their host genes. (**A**) Volcano plot showing the relative expression change of 514 miRNAs between patients from the short-lived and long-lived subgroup. miRNAs with a significant change in expression (q < 0.1) are colored, others in gray. (**B**) Correlation of miRNA and corresponding host gene expression values across 42 *DNMT3A*-mutated patients. Pearson correlation coefficients were set to zero (dashed vertical line, yellow coloring) for miRNAs without a protein-coding host gene. For both figure panels, triangles indicate miRNAs that show (white) or do not show (turquoise) a significant coexpression (positive correlation) with their respective host gene (q < 0.1).

**Figure 4:**
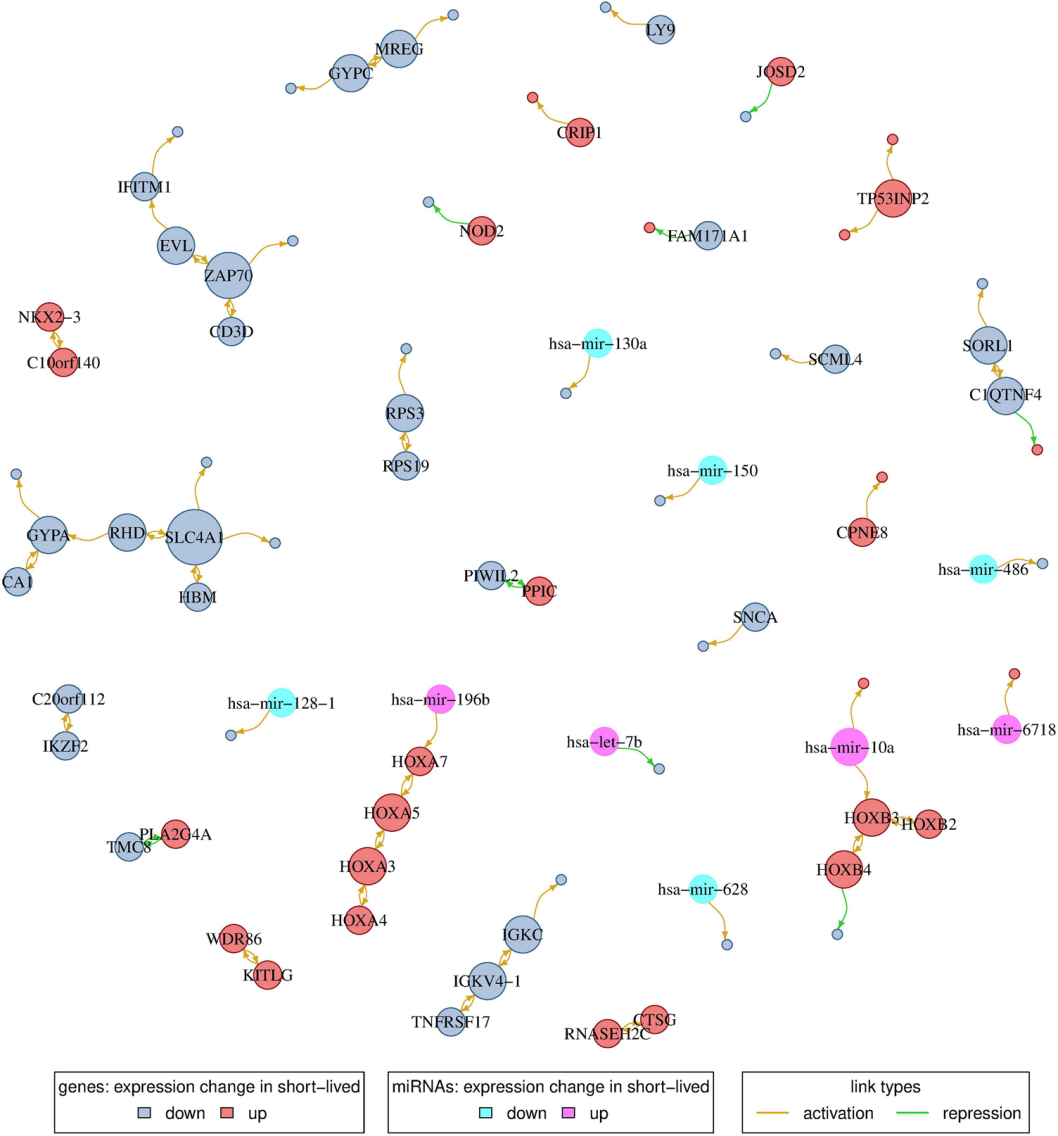
Gene and miRNA regulatory network. Nodes represent either genes that are differentially expressed between the two patient subgroups or miRNAs selected as predictors during network inference. Nodes are colored according to whether a gene/miRNA shows an increase or decrease in expression in the short-lived relative to the long-lived patient subgroup. Gene/miRNA names are shown for putative regulator nodes (out-degree > 0) with node sizes being proportional to their out-degree. Potential activating and repressing links are shown in yellow and green color, respectively; only links present in at least two-thirds of the networks were considered. Note that links can represent direct or indirect regulatory dependencies or may only represent correlations.

### A miRNA expression signature discriminates short- and long-lived *DNMT3A*-mutated patients

To further analyze differences between the short- and the long-lived subgroup with respect to gene regulation, we considered miRNA expression data from TCGA available for 42 of the 51 *DNMT3A*-mutated AML patients. As for the gene expression data, we conducted a differential expression analysis and identified 25 differentially expressed miRNAs discriminating patients from the two subgroups using a q-value cut-off of 0.1 (Fig. 3A, Supplementary Table 3).

Interestingly, the relative fractions of up- and downregulated miRNAs in the short-lived compared to the long-lived subgroup were highly uneven. The large majority of miRNAs (21 out of 25) were downregulated in the short-lived subgroup, while only four miRNAs were upregulated. An altered miRNA expression can have different reasons: (i) it could be caused by the altered expression of a host gene that contains the affected miRNA, or (ii) the expression of a miRNA can be altered directly and independent of its host gene or in the absence of a host gene (e.g. a miRNA encoded in an intergenic chromosomal region). Therefore, we tested whether or not the expression of a miRNA is significantly correlated with the expression of its host gene across all *DNMT3A*-mutant patients.

Based on this correlation analysis, we found that the first category, e.g. miRNAs with significant host gene expression correlation, contained 6 of the 25 differentially expressed miRNAs (Fig. 3B). The expression of *miR-199a-2*, whose gene co-localizes with the dynamin gene *DNM3*, was positively correlated with *DNM3* expression (r = 0.432, P = 0.004). Also the expression of *miR-3154* and *miR-199a-1*, which co-localize with the other two dynamin genes, were positively correlated with the expression of their host genes (*miR-3154* vs. *DNM1*: r = 0.390, P = 0.011; *miR-199a-1* vs. *DNM2*: r = 0.266, P = 0.089), although not statistically significant after correction for multiple testing (i.e., q > 0.1). Comparing short- to long-lived patients, the expression of *DNM1* and *DNM3* was moderately decreased, whereas the expression of *DNM2* did not differ between both subgroups (Supplementary Table 3). The other five miRNAs that had significantly positive expression correlations with their host genes were *miR-10a* (host gene *HOXB3*), *miR-126* (*EGFL7*), *miR-362* (*CLCN5*), *miR-26a-1* (*CTDSPL*) and *miR-551b* (*EGFEM1P*).

The second category contained 19 of 25 differentially expressed miRNAs that did not show coexpression with their host genes or are encoded in inter-genic regions and do not have a host gene (Fig. 3B). An association with AML has been reported previously for 13 of them (Supplementary Table 4). For instance, *miR-181a-2*, *miR-181b-2* and *miR-30a* are known to be associated with a favorable prognosis upon up-regulation of their expression^19,37^, which is in line with a strong down-regulation of these three miRNAs in the short-lived relative to the long-lived subgroup. Similarly, we could reconfirm an up-regulation of *let-7b* in the context of *NPM1* mutations and a down-regulation of *miR-130a* in the context of *FLT3* mutations^37^ in the short-lived subgroup. Further, we found a down-regulation of *miR-331* in the short-lived subgroup, which differs from^19^ reporting that the up-regulation of *miR-331* was associated with poor prognosis. We also observed decreased expression of *miR-98* in the short-lived subgroup, which differs from previous findings that *miR-98* is up-regulated in the background of *NPM1* mutations (Supplementary Table 4). In addition, no direct associations with AML have been reported so far for the 4 miRNAs (*miR-153-2, miR-3065, miR-6718, miR-95*) (Supplementary Table 4), but associations with other types of cancer suggest that differences in their expression between short- and long-lived *DNMT3A*-mutant AML patients could also be important for prognosis (see “Discussion”).

### Regulatory networks reveal potential molecular major regulators distinguishing short- from long-lived *DNMT3A*-mutated patients

In order to investigate the combined effect of gene and miRNA expression on gene regulation we integrated these two types of data using a regulatory network-based approach. We started by reconstructing a signature gene-specific network to reveal potential regulators that distinguish short- from long-lived patients. Considering the 260 differentially expressed genes observed between both groups (Fig. 2A, Supplementary Table 3), we modeled the expression of a signature gene as a linear combination of the expression levels of the other 259 signature genes distinguishing short- from long-lived patients (see “Methods” for details). The prediction of robust links between genes during reconstruction of the network was complicated due to the small number of *DNMT3A*-mutated patients. Therefore, we repeated the network inference 100 times with different, randomly selected training sets of patients to identify network links that robustly occurred in at least two-thirds of the networks with a q-value of 0.1 or smaller. This enabled to predict the expression values of on average 18.3% of the 260 signature genes. In a second step, we further added the expression values of all 514 miRNAs as additional predictors to the network model and repeated the analysis. This slightly improved the fraction of signature genes with predictable expression levels to 21.9%. The prediction accuracy of those genes, quantified by computing correlation coefficients between measured and predicted expression levels on the network-specific test sets, was high and significantly shifted into the positive range (mean correlation: 0.805, Wilcoxon signed rank test: P < 0.0001, Supplementary Fig. 5).

The resulting consensus network included 76 genes and 9 miRNAs (Fig. 4, Supplementary Table 5). This network consisted of several modules that were composed of two to eight genes. Two of the larger network modules were up-regulated in the short-lived subgroup and contained, respectively, four *HOXA* and three *HOXB* genes which are well-known major regulators of cell development and that have frequently been reported to be dysregulated in cancers including AML^38–40^. Six additional network modules with at least three genes and their components are summarized in Table 2. Each of the potential regulators in these network modules (labeled nodes in Fig. 4) was down-regulated in the short-lived compared to the long-lived subgroup. Interestingly, two of the six modules (network modules 1 and 4) contained genes that code for proteins expressed in erythrocytes or other blood components (*HBM, RHD, GYPA, GYPC, CA1*), or have been implicated in blood-associated diseases like anemia (*GYPA*) (Table 2). In addition, *RPS19* of network module 6, encoding a ribosomal protein, has been linked to anemia, too (Table 2). Genes of the remaining three modules (network modules 2, 3, and 5) are involved in innate or adaptive immunity (*ZAP70, CD3D, IFITM1, TNFRSF17, IGKV4-1, IGKC, C1QTNF4*) (Table 2).

We further analyzed the protein-coding genes that were directly connected to one of the nine miRNAs in the network representing potential targets for miRNA-based post-transcriptional regulation (Fig. 4). Among them were five genes with known transcription factor activity (*PBX3, HOXB3, LEF1, HOXA7, LBH*) and three genes with oncogenic potential (*PAPD7, PBX3, LEF1*) for which a role in other cancers has been suggested previously (Table 3). Interestingly, a role during leukemogenesis and/or implications for clinical prognosis in AML has been reported for eight of the nine miRNAs (Supplementary Table 4). This included the differential regulation of *let-7b* and *miR-130a* already mentioned above as well as of *miR-10a* and *miR-486* in the context of *NPM1* or *FLT3* mutations, effects on prognosis upon differential regulation of *miR-128-1* and *miR-150*, an increased cell survival and proliferation prompted by expression changes of *miR-196b* targeting *HOXB8*, and regulation of *miR-628* by cytokines^18,19,37,41,42^.

**Figure 5:**
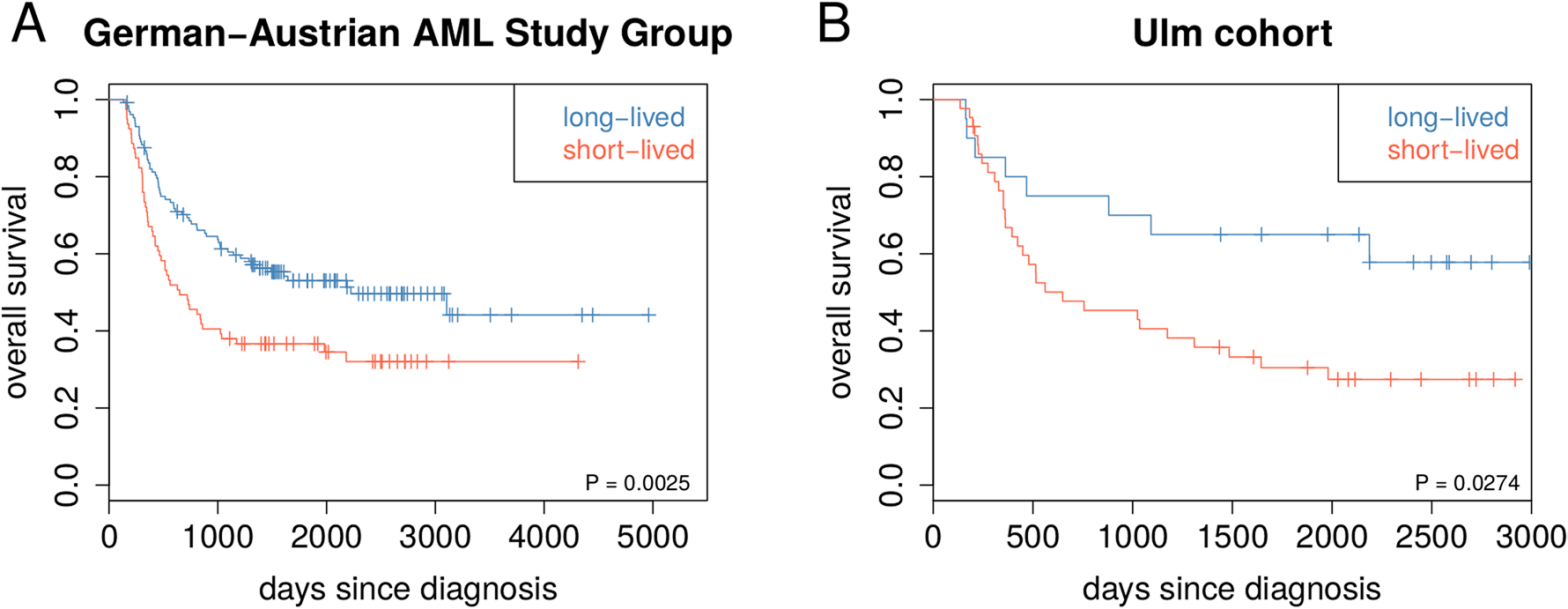
Validation based on independent *DNMT3A*-mutant AML patients. (**A**) Gene mutation based validation. Kaplan-Meier curves for an independent cohort of 208 *DNMT3A*-mutated AML with bone marrow transplantation from the German-Austrian AML Study Group. For each of these patients, the most similar *DNMT3A*-mutated AML patient of the TCGA cohort was determined by counting mismatches between the corresponding gene mutation profiles. Each patient was assigned to the short-lived or to the long-lived group based on the class label of the most similar TCGA patient (short-lived: red, 79 patients; long-lived: blue, 129 patients). Log-rank test for short- vs. long-lived: P < 0.003. (**B**) Gene expression based validation. Kaplan-Meier curves for an independent cohort of 63 *DNMT3A*-mutated AML patients from the University Hospital of Ulm that were also part of the German-Austrian AML Study Group. For each of these patients, correlations between its signature gene expression profile with the average short-lived and long-lived signature gene expression profiles of the *DNMT3A*-mutated AML patients from TCGA were computed. Each patient was assigned to the short-lived or to the long-lived group based on the maximum of both correlations (short-lived: red, 43 patients; long-lived: blue, 20 patients). Log-rank test for short- vs. long-lived: P < 0.03.

**Figure 6:**
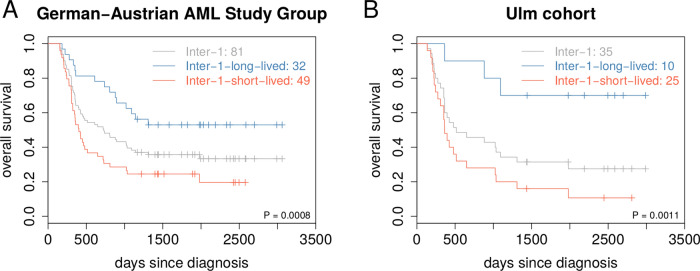
Improvement of ELN 2010 risk classification by additional short- and long-lived stratification. (**A**) Gene mutation-based validation of the 81 independent validation patients from the German-Austrian AML Study Group of the ELN 2010 risk category intermediate-1 (Inter-1). For each of these patients, the most similar *DNMT3A*-mutated AML patient of the TCGA cohort was determined by counting mismatches between the corresponding gene mutation profiles. Each patient was assigned to the short-lived or to the long-lived subgroup based on the class label of the most similar TCGA patient. Kaplan-Meier curves of this additional stratification are shown in red for the 49 Inter-1-short-lived patients and in blue for the 32 Inter-1-long-lived patients. The basic Kaplan-Meier curve without additional stratification of these patients is shown in grey. Log-rank test for Inter-1-short- vs. Inter-1-long-lived: P = 0.0008. A global overview of the additional stratification of all ELN 2010 risk categories is shown in Supplementary Fig. 7. (**B**) Gene expression based validation of the 35 independent validation patients from the Ulm cohort of the ELN 2010 risk category intermediate-1 (Inter-1). For each of these patients, correlations between its signature gene expression profile with the average short-lived and long-lived signature gene expression profiles of the *DNMT3A*-mutated AML patients from TCGA were computed. Each patient was assigned to the short-lived or to the long-lived subgroup based on the maximum of both correlations. Kaplan-Meier curves of this additional stratification are shown in red for the 25 Inter-1-short-lived patients and in blue for the 10 Inter-1-long-lived patients. The basic Kaplan-Meier curve without additional stratification of these patients is shown in grey. Log-rank test for Inter-1-short- vs. Inter-1-long-lived: P = 0.0011. A global overview of the additional stratification of all ELN 2010 risk categories is shown in Supplementary Fig. 8.

### Validation based on independent *DNMT3A*-mutant AML patients

We considered gene mutation and gene expression data of independent *DNMT3A*-mutated AML patients from the German-Austrian AML Study Group^34,43–45^ to analyze whether the characteristic gene mutation and expression profiles that distinguished short- and long-lived *DNMT3A*-mutated TCGA AML patients are also of potential prognostic relevance for other patients.

To analyze the transferability of the prognostic relevance of our initial grouping of gene mutation profiles of *DNMT3A*-mutated TCGA AML patients into a short- and long-lived subgroup (Fig. 1A, Supplementary Table 1), we considered gene mutation data of 208 *DNMT3A*-mutant AML patients from the German-Austrian AML Study Group that were initially treated in a similar manner followed by a bone marrow transplantation. We determined for each of these new patients the most similar *DNMT3A*-mutated TCGA AML patient and assigned its corresponding label (short- or long-lived) to the new patient. We found that the gene mutation profiles of short- and long-lived *DNMT3A*-mutated TCGA AML patients enabled to separate the 208 *DNMT3A*-mutant AML patients from the German-Austrian AML Study Group into a short- and long-lived subgroup that differed significantly in survival (Fig. 5A, log-rank test: P < 0.003).

In addition, we also analyzed the transferability of the prognostic relevance of the characteristic gene expression signature that distinguished short- and long-lived *DNMT3A*-mutated TCGA AML patients (Fig. 2A, Supplementary Table 2, q < 0.1). We therefore considered gene expression data of 63 *DNMT3A*-mutant AML patients from the University Hospital of Ulm that were also part of two clinical trials of the German-Austrian AML Study Group^44, 45^. The majority of these patients received a bone marrow transplantation (47 of 63). We determined for each of these new patients the similarity to the TCGA-based short- and long-lived signature and assigned to each patient the label of the most similar class (short- or long-lived). We found that the characteristic gene expression signature that distinguished short- and long-lived *DNMT3A*-mutated TCGA AML patients also enabled to separate the 63 *DNMT3A*-mutant AML patients from the University Hospital of Ulm into a short- and long-lived subgroup that differed significantly in survival (Fig. 5B, log-rank test: P < 0.03). This separation significance was further improved when we only considered the 47 patients that received a bone marrow transplantation (log-rank test: P < 0.016).

Further, we also analyzed if our short- and long-lived classification of *DNMT3A*-mutant AML patients can help to improve the widely considered European LeukemiaNet (ELN) prognostic scoring systems^46,47^. Risk classifications according to the ELN 2010 system^46^ were publicly available for 192 of 208 patients of the German-Austrian AML Study Group considered for the gene mutation-based validation^34^. Our additional stratification into short- and long-lived patients significantly improved the risk stratification of patients of the ELN 2010 intermediate-1 risk category (Fig. 6A, log-rank test: P = 0.0008). Also patients of the ELN 2010 adverse risk group could potentially benefit from our additional stratification (Supplementary Fig. 7). Further, our additional stratification had no impact on the stratification of patients of the ELN 2010 intermediate-2 or favorable risk categories (Supplementary Fig. 7). We also analyzed the impact of our short- and long-lived stratification on the revised ELN 2017 risk classification^47^. This was possible for 134 of 208 patients, but excluded patients with a *FLT3*-ITD mutation, because the *FLT3*-ITD-to-wild-type allelic ratios required for a reclassification were not publicly available^48^. In this limited analysis, we found that our additional stratification had no impact on the ELN 2017 favorable risk category, but there were too few patients to interpret the additional stratification of the other risk categories (Supplementary Fig. 9A-B).

In addition, the ELN 2010 risk classification was also available for 62 of 63 patients of the Ulm cohort considered for the gene expression-based validation^34^. We found that our additional stratification into short- and long-lived patients again significantly improved the risk stratification for patients of the ELN 2010 intermediate-1 risk category (Fig. 6B, log-rank test: P = 0.0011). Interestingly, there was also a clear tendency that patients of the ELN 2010 favorable risk category could potentially benefit from our additional stratification (Supplementary Fig. 8). Further, our additional stratification had no impact on patients of the ELN 2010 intermediate-2 or adverse risk categories (Supplementary Fig. 8). We also analyzed the impact of our additional stratification on the revised ELN 2017 risk classification that was available for 37 of 63 patients^48^. We observed that patients of the ELN 2017 favorable risk group can potentially benefit from our additional stratification (Supplementary Fig. 9C,D). Similar trends were also present for the ELN 2017 intermediate and adverse risk categories. However, there were too few patients within the different ELN 2017 risk categories to analyze the significance of these trends.

Nevertheless, all these results round off our different computational studies for the TCGA cohort and indicate that the characteristic discriminative gene mutation and expression signatures that distinguished short- from long-lived *DNMT3A*-mutated TCGA AML patients are also predictive for other independent patient cohorts and potentially useful to improve patient stratification.

## Discussion

A somatic mutation of *DNMT3A* occurs in about one fourth of adult AML cases. Mutations of this gene have frequently been associated with poor survival^9,14,25,30,31^, but also substantially longer survival or long-term remissions have been reported for some *DNMT3A*-mutant AML patients^32,33^. Detailed molecular differences that may contribute to these survival differences have not been characterized so far. This motivated us to analyze all *DNMT3A*-mutant patients of the TCGA AML cohort with the help of well-established computational tools. We identified two robust subgroups of *DNMT3A*-mutant patients purely based on clustering of somatic gene mutation profiles and further found that both subgroups showed significant survival differences.

Further comparisons showed that the short-lived subgroup had a strong enrichment of mutations of the R882 codon of the catalytic methyltransferase domain of *DNMT3A*, whereas the number of R882 and non-R882 mutations was nearly equal within the long-lived subgroup. This mutation type-specific effect on prognosis has been noted before^25^, but was not sufficient for a full discrimination of our two subgroups. Thus, additional molecular factors are likely to contribute to the observed survival differences.

Mutated *DNMT3A* has been shown to induce genomic instability in a human leukemic cell line model^49^. We therefore compared the short- and long-lived subgroup in terms of mutated genes and cytogenetic rearrangements. Interestingly, the number of mutated genes was significantly smaller in the short-lived subgroup. In addition, the majority of patients of both subgroups had normal cytogenetic profiles, but especially some patients of the long-lived subgroup showed duplications or rearrangements of chromosome 8 that have not been observed within the short-lived subgroup. Thus, the overall shorter survival of patients in the short-lived group cannot be explained by a greater mutational burden or increased rates of abnormal cytogenetic profiles.

We found *NPM1* and/or *FLT3* mutations in every short-lived patient but only in few long-lived patients. This overrepresentation of *NPM1* and/or *FLT3* mutations in the short-lived subgroup is not unexpected, because *DNMT3A*, *NPM1*, and *FLT3* are the most frequently mutated genes found in AML^10^. The co-occurrence of mutations of all three genes has previously been suggested to define a specific subtype of AML with unique epigenetic features^10^ and frequent mutations of *NPM1* and *FLT3* in *DNMT3A*-mutant patients have also been observed in other AML studies^9,25,50^. Importantly, *NPM1* and *FLT3* are both established prognostic markers in routine clinical practice^47^. *FLT3* mutations (ITD: internal tandem duplication of the juxtamembrane region, TKD: point mutations in the second tyrosine kinase domain) have been associated with increased relapse risk and poor outcome of AML patients^51,52^. The frequency of *FLT3-ITD* and *FLT3*-TKD mutations was nearly identical in the short-lived subgroup and an additional stratification according to the specific type of *FLT3* mutation did not further improve our classification of *DNMT3A*-mutant AML patients from TCGA. *NPM1* mutations frequently co-occur together with *FLT3*-ITD mutations, which counteracts a favorable prognosis that is observed for AML patients that only have a *NPM1* mutation but no *FLT3* mutation^12,47,53^. This is also supported by our two subgroups. The majority of short-lived patients had co-mutations of *NPM1* and *FLT3*, whereas long-lived patients did not show *NPM1* mutations in the background of *FLT3* mutations. Thus, our study clearly indicates that *NPM1* and/or *FLT3* mutations are likely to contribute to the prognosis of *DNMT3A*-mutant patients. This is supported by the previous findings that *DNMT3A* mutations jointly act with *FLT3* and *NPM1* mutations to promote resistance to anthracycline chemotherapy^54^ and that concurrent mutations of *DNMT3A*, *FLT3*, and *NPM1* have also been associated with poor prognosis of AML patients^55^. In addition, all our survival analyses in combination with the presence or absence of *DNMT3A* mutations further support that *DNMT3A* mutations have an additional negative impact on survival that is independent of *FLT3* and/or *NPM1* mutations or co-mutations of both genes. This is supported by findings for the presence or absence of *DNMT3A* mutations in AML patients with *FLT3* mutations^50^. Additional experiments should be done to elucidate whether the *DNMT3A* mutation cooperates with *FLT3* and *NPM1* co-mutations.

Since an increased rate of *DNMT3A*-R882 mutations was observed for our short-lived subgroup, we also analyzed a large independent cohort of AML patients^34^ and observed an enrichment of *DNMT3A*-R882 and *NPM1* co-mutations and an enrichment of concurrent *DNMT3A*-R882, *NPM1*, and *FLT3* mutations compared to AML patients with *DNMT3A* mutations that did not affect the R882 codon. Interestingly, the blood composition of these groups differed in dependency of the type of the *DNMT3A* mutation indicating an impact on the differentiation capabilities of AML cells. Additional experiments are required to validate the accumulation of *NPM1* and/or *FLT3* mutations and to analyze the differentiation capabilities of AML cells in the background of specific *DNMT3A* mutations.

We further compared the gene expression profiles of the short- and long-lived subgroup revealing a molecular signature of 260 protein-coding genes that distinguished both subgroups. This signature included many transcription factors and genes of cancer-associated pathways like p53, VEGF and PI3K-Akt signaling and DNA replication. Importantly, a clustering of the patients based on these signature genes largely recapitulated the short-lived and long-lived subgroup and further revealed a set of patients with mixed expression levels. This indicates that at least three different transcriptional programs are associated with survival differences of *DNMT3A*-mutant AML patients. Further, it is important to note that *NPM1* or *FLT3* mutations or co-mutations of both genes that were observed for each short-lived patient also contribute to the observed expression differences. Therefore, our comparison of short- and long-lived gene expression profiles does not allow to disentangle the individual contributions of *DNMT3A*, *FLT3*, or *NPM1* mutations. Still, all our survival analyses comparing the presence or absence of *DNMT3A* mutations in the background of *NPM1* and/or *FLT3* mutations suggest an additional contribution of *DNMT3A* mutations. This additional contribution is also included in the gene expression signature and further supported by our gene expression-based classification of independent *DNMT3A*-mutant AML patients.

Alterations of miRNA expression profiles play an important role in AML^18,19^. We therefore compared the miRNA expression profiles of the short- and long-lived subgroup. We revealed a dominant trend of miRNA downregulation in the short-lived subgroup suggesting a wide-spread activation of otherwise repressed protein-coding genes, including known AML oncogenes and other oncogenes that were not associated with AML before. Further, associations with AML prognosis and/or mutation of *NPM1* and *FLT3* have already been reported for most miRNAs, but we also identified four miRNAs that have not been reported for AML so far. This included three miRNAs that were downregulated in the short-lived subgroup (i) *miR-153-2* implicated in brain, lung, liver and epithelial cancers^56–59^, (ii) *miR-3065* for which an association with altered gene expression regulation in breast tumors was suggested^60^, and (iii) *miR-95* known to be differentially expressed in different human cancers^61–63^ with shown impacts on cell proliferation, invasion, migration, and apoptosis in a pancreatic tumor cell line and in hepatocellular carcinoma^61,63^. We did not find cancer-associated reports for the fourth miRNA *miR-6718*, but its strong 2.6-fold upregulation in the short-lived subgroup and the selection by our regulatory network approach suggests an association with prognosis. In addition, we discovered a downregulation of all three dynamin genes in the short-lived subgroup based on their co-localized miRNAs. This may have an impact on endocytosis, asymmetric cell divisions, and blockage of immune signals^64–67^. This suggests that these miRNAs could represent important biomarker candidates to discriminate between short- and long-lived *DNMT3A*-mutant AML patients. Additional experimental studies should be done to validate these potential markers and to better understand how they alter molecular mechanisms in *DNMT3A*-mutant AML patients.

We also learned gene regulatory networks to identify potential major regulators and to delineate modules of protein-coding and miRNA genes that were altered between the short- and long-lived subgroup. Due to the relatively small number of AML patients with *DNMT3A* mutations, our consensus network contained only relatively few genes compared to networks from similar studies of other cancers^68,69^. Still, those genes present in the network and the links between them were inferred with high confidence. It is important to note that the inferred links between genes can reflect direct or indirect regulatory dependencies or only represent correlations, because our network reconstruction method is based on correlations between gene expression levels. Yet, larger sub-networks can still point toward cellular pathways that are altered between both subgroups. Our revealed modules suggest alterations of several cellular processes in short-lived relative to long-lived patients. This included genes of the PI3K-Akt and p53 signaling pathway involved in AML^70,71^ and an upregulation of HOX genes altered in leukemia^38,40^. In addition, we also identified genes that are expressed in different blood components. This included three genes downregulated in the short-lived subgroup - *SLC4A1*, *GYPA* and *RPS19* - that have previously been associated with anemia^72–74^. Notably, *SLC4A1* and its co-factor *GYPA* play a major role in oxygen and carbon dioxide exchange in erythrocytes^75,76^ and their downregulation in the short-lived subgroup could be associated with less differentiated leukemic cells. Further, we found three gene modules with immunity-related functions downregulated in the short-lived subgroup and an increased number of differentially expressed cytokine receptor signaling pathway genes suggesting that immune evasion might be more effective in the short-lived subgroup, but immunosuppression in AML is still poorly understood^77^. The identified putative major regulators potentially represent important candidates for the development of biomarkers that could distinguish between short- and long-lived patients. Additional experimental validation studies are required to test their prognostic potential and to further characterize their functional role in *DNMT3A*-mutant AML patients.

Moreover, we also showed that the characteristic gene mutation and expression signatures that distinguished short- from long-lived *DNMT3A*-mutant TCGA AML patients contain relevant information that can be used to classify other independent *DNMT3A*-mutant AML patients as short- or long-lived. We demonstrated this for *DNMT3A*-mutant AML patients from the German-Austrian AML Study Group. Thus, our revealed molecular signatures could potentially provide a useful basis to enable a better stratification of *DNMT3A*-mutant AML patients to more precisely identify patients that are of high risk for a fast relapse. This is also supported by the interpretation of our results with respect to the cytogenetic and molecular risk classification provided by TCGA, which assigned more than 82% of the *DNMT3A*-mutant patients to the intermediate risk group, whereas the remaining patients were assigned to the poor risk group, except one unclassified patient. Since our approach significantly improved the stratification of these TCGA patients, this also clearly indicates that our approach can improve this cytogenetic and molecular risk classification. The value of our approach is further supported by the significant improvement of the stratification of patients that were assigned to intermediate-1 risk category according to the ELN 2010 prognostic scoring system^46^. Further, we also observed potential benefits of our additional stratification for the ELN 2010 risk categories favorable and adverse, but more patients would have been required for a robust significance analysis. In addition, an analysis of the revised ELN 2017 risk categories^47^ indicated that the favorable and intermediate risk groups could potentially benefit from our additional stratification, but this should be taken with caution, because this analysis was only possible for a subset of our validation patients. Additional validation studies are necessary to analyze how our findings generalize to other patient cohorts and how they impact on patient outcome. Future studies should include an extended comparison to the revised ELN 2017 scoring system. This was only partly possible in our study, because molecular data such as the *FLT3*-ITD-to-wild-type allelic ratio required for a reclassification were not publicly available for the patients considered in our study. However, a recent study has shown that *DNMT3A*-mutant AML patients have a worse prognosis than *DNMT3A* wild type patients for individual ELN 2017 risk categories^48^. Our study indicates that an improved stratification of individual risk categories might even be possible within the group of *DNMT3A*-mutant AML patients.

Our study represents the first in-depth computational approach to identify molecular factors associated with survival differences of *DNMT3A*-mutant AML patients. This may provide a basis to develop molecular markers for improved patient stratification. Future studies are required to further analyze and validate the findings of our computational study.

## Methods

### Molecular data

Gene and miRNA expression data and somatic mutations of patients from the TCGA AML cohort were obtained from the TCGA data portal (gdc.cancer.gov). After excluding lowly expressed genes with a counts per million value smaller one in two-thirds or more of the patients, we normalized the raw expression data using the R/Bioconductor package *limma* with normalization method cyclic loess^78^. By using information on the *DNMT3A* mutational status from the somatic mutation data, we determined 51 *DNMT3A*-mutated AML patients and derived corresponding gene expression (47 of 51 patients, 15,623 genes) and miRNA (42 of 51 patients, 514 miRNAs) data sets. Details to *DNMT3A*-mutations and processed data sets are provided in Supplementary Table 1.

### Clustering based on somatic mutation data

We considered each of the 51 AML patients with a *DNMT3A* mutation and created for each patient its binary gene mutation profile by setting the entry of each gene to one (mutated) or to zero (not mutated) in dependency of the patient-specific gene mutation status. Next, we performed a hierarchical clustering of tumors based on binary profiles of the somatic mutation data using R with 1 minus Pearson correlation as distance measure with distances ranging from zero (two completely identical mutation profiles) to one (two completely different mutation profiles) in combination with Ward’s clustering method (*ward.D2*)^79^. Note that the Pearson correlation coefficient of two binary variables is equal to the phi coefficient^80^. Hierarchical clustering initially considers each patient as a separate cluster and then repeats the following two steps until all clusters are merged together: (i) identification of the two clusters with the smallest distance followed by (ii) merging of these two clusters into a joint cluster. These iterative merging steps enable to reveal the hierarchical relationships between the clusters that are stored in a tree-structure called dendrogram. Two tumor subgroups were derived by cutting the resulting clustering dendrogram into two sub-trees. These subgroups were named ’short-lived’ and ’long-lived’ according to survival differences between the subgroups (see below). The TCGA identifiers for patients of the short- and long-lived subgroup are provided in Supplementary Table 2. To assess the robustness of this patient clustering, we excluded *k* randomly selected patients, repeated the clustering into two groups as described above, and performed a log-rank test for survival differences between the groups (see below). We tested $k = 2, 4, 6, 8, 10$, and repeated the analysis 10,000 times for each *k*. We did not test larger values of *k* owing to the relatively small number of *DNMT3A*-mutated AML patients in the data set.

### Survival analysis

Information about days to death (for patients with status ’Dead’) or days to last follow-up (for patients with status ’Alive’) was taken from the TCGA clinical data (Supplementary Table 2). Last follow-up events were considered as non-informative censoring events. We generated survival curves and performed log-rank tests using the R package *survival*^81^.

### Identification of differentially expressed genes and miRNAs

Differential gene and miRNA expression analysis between the short- and long-lived subgroup was done following *limma’s* standard workflow^78^. Results of the gene and miRNA expression analysis are provided in Supplementary Table 3. Differentially expressed (signature) genes or miRNAs were selected using an FDR-adjusted p-value (q-value) cut-off of 0.1.

### Gene and pathway annotation enrichment analysis

Gene, signaling pathway, and metabolome annotations were obtained from^35^. The number of signature genes per annotation category was counted separately for up- and downregulated genes and their significance of enrichment per category was calculated using Fisher’s exact test.

### Signature-specific regulatory network inference

We inferred transcriptional regulatory networks that model the expression of a signature gene as a linear combination of weighted expression values of the other signature genes and, optionally, of miRNAs. Mathematical details to the underlying linear model are provided in^35,82^. This approach has further been applied in similar studies of other human cancers^68,69,83,84^. We learned two types of networks using (i) the expression values of signature genes and (ii) the expression values of signature genes and miRNAs as predictors. miRNA expression values were set to zero for patients without available miRNA profiles. Lasso regression^85^ in combination with a significance test for lasso^86^ were used to estimate the coefficients and their corresponding significance of the predictors for each signature gene-specific linear model^82^. This sparse regression approach selects the most relevant predictors that best explain the observed expression levels of a signature gene across the *DNMT3A*-mutant AML patients.

Both network approaches were validated through cross-validation by repeated random sub-sampling. To this end, the data was randomly partitioned into a training set constituting three-quarter of the *DNMT3A*-mutated AML patients and a test set constituting the remaining one-fourth of patients. A network was constructed on the training data, and the expression of the signature genes was predicted and compared to the experimentally measured expression for the test data. This procedure was repeated 100 times. To assess prediction accuracy, we calculated Pearson correlation coefficients of predicted and measured gene expression averaged over the 100 networks. A consensus network was constructed by including all links with q-values of 0.1 or smaller that were predicted in at least two-thirds of the 100 networks.

### Validation based on independent *DNMT3A*-mutant AML patients

To validate the separation capability of the characteristic gene mutation profiles of short- and long-lived *DNMT3A*-mutant AML patients from TCGA, we downloaded publicly available gene mutation profiles and clinical data of AML patients from https://github.com/gerstung-lab/AML-multistage/tree/master/data^34^. We considered all 208 *DNMT3A*-mutated AML patients from the German-Austrian AML Study Group (AMLSG)^43–45^ that received a bone marrow transplantation to obtain a large validation cohort of patients that were treated similarly. The majority of these patients (204 of 208) were part of two clinical trials (AMLHD98A: 77^44^; AMLSG0704: 127^45^) focusing on AML patients in the age range of 18 to 65. The other four patients were part of the AMLHD98B trial that considered AML patients of age 61 or older^43^. Considered patients from AMLHD98A received an induction chemotherapy with idarubicin, cytarabine and etoposide (ICE) followed by allogeneic transplants. Treatment of considered patients form AMLSG0704 and AMLHD98B was similar, but patients were randomly assigned to receive ICE or ICE plus all-trans retinoic acid (ATRA) as induction therapy before transplantation^12^. We computed the most similar *DNMT3A*-mutated TCGA AML patient for each of these 208 patients by counting mismatches between each corresponding pair of gene mutation profiles. We had to focus on 31 genes that overlapped with the mutated genes of *DNMT3A*-mutated TCGA AML patients, because the data from^34^ was obtained by targeted sequencing of selected cancer genes. We assigned each of the 208 patients either to the short- or to the long-lived group based on the class label of the most similar TCGA patient and performed a survival analysis as described in the section ’Survival analysis’ above (Supplementary Table 6). Further, we also considered the European LeukemiaNet (ELN) 2010 risk classification^46^ available for 192 of 208 patients to analyze if an additional stratification of each individual ELN 2010 risk category based on our short- and long-lived classification can improve this prognostic scoring system (Supplementary Table 6). We realized this by an extended survival analysis for the patients of an individual risk category in comparison to our corresponding short- and long-lived classifications of these patients. Similarly, we also analyzed our stratification into short- and long-lived patients considering the revised ELN 2017 risk classification^47^. This was only possible for 134 of 208 validation patients that were reclassified in^48^ (Supplementary Table 6). The other validation patients could not be considered, because *FLT3*-ITD-to-wild-type allelic ratios required for a reclassification were not publicly available.

To validate the separation capability of the gene expression signature of short- and long-lived *DNMT3A*-mutant AML patients from TCGA, we considered a cohort of 218 AML patients from the University Hospital of Ulm of which 63 had a *DNMT3A* mutation. The majority of these 63 patients were part of the AMLSG0704 clinical trial^45^ (59) and the remaining 4 patients were part of the AMLHD98A clinical trial^44^ of the German-Austrian AML Study Group. The majority of these patients received a bone marrow transplantation (47 of 63). The AML gene expression profiles of these patients were measured on Affymetrix HG-U133 Plus 2 microarrays. We normalized the gene expression data set using GCRMA^87^ in combination with a BrainArray design file (HGU133Plus2_Hs_ENTREZG 15.0.0). We focused on the 257 signature genes of the 260 signature genes from our TCGA analysis (Fig. 2A, Supplementary Table 3, q < 0.1) that were measured on the Affymetrix arrays. We computed for each of the 63 *DNMT3A*-mutated patients rank-based correlations (Kendall’s tau) between its signature gene expression profile and the average short-lived and long-lived signature gene expression profiles of the *DNMT3A*-mutated AML patients from TCGA. We assigned each patient either to the short-lived or to the long-lived group based on the maximum of both correlations and performed a survival analysis as described above (Supplementary Table 7). We also repeated this analysis only focusing on the 47 patients that received a bone marrow transplantation. Further, we again considered the ELN 2010 risk classification^46^ available for 62 of 63 patients (Supplementary Table 7) and performed an additional survival analysis to analyze if our short- and long-lived classification can improve the individual risk categories. Similarly, we analyzed our short- and long-lived stratification considering the revised ELN 2017 risk classification^47^ for the subset of 37 of 63 validation patients that could be reclassified in^48^ (Supplementary Table 7).

### Ethical approval and informed consent

Not applicable. No ethical approval was required for this study. All utilized public omics data sets were generated by others who obtained ethical approval.

## Supplementary information

Supplementary Figures

Supplementary Table 1: Somatic mutation and normalized gene and miRNA expression data of the analyzed DNMT3A-mutated AML patients.

Supplementary Table 2: Survival data and gene expression groups of analyzed AML patients.

Supplementary Table 3: Limma results of differential gene and miRNA expression analysis.

Supplementary Table 4: Differentially expressed miRNAs and miRNAs part of the regulatory network and their possible roles in leukemias.

Supplementary Table 5: Links present in the regulatory network.

Supplementary Table 6: Gene mutation based survival analysis of DNMT3A-mutant patients from the German-Austrian AML Study Group.

Supplementary Table 7: Gene expression based survival analysis of DNMT3A-mutant AML patients form the University Hospital of Ulm.

## Data Availability

Molecular data and meta-information of all considered TCGA AML patients are publicly available from The Genomic Data Commons Data Portal (https://portal.gdc.cancer.gov/). Additional files attached to this manuscript contain considered molecular data, survival information, and learned network links. Basic implementations of the algorithms considered for network inference are publicly available from GitHub (https://github.com/seifemi/regNet).
