# Supplementary material for: Survival differences and associated molecular signatures of DNMT3A-mutant acute myeloid leukemia patients
Source: Sci Rep. 2020 Jul 29;10:12761. doi: 10.1038/s41598-020-69691-8 (PMC7391693; doi:10.1038/s41598-020-69691-8)
Supplement: Supplementary file 1 — Supplementary Figures [file 41598_2020_69691_MOESM1_ESM.pdf]

Survival differences and associated molecular signatures of *DNMT3A*-mutant acute myeloid leukemia patients

Chris Lauber[1], Nádia Correia[2], Andreas Trumpp[2], Michael A. Rieger[3], Anna Dolnik[4], Lars Bullinger[4], Ingo Roeder[1,5], Michael Seifert[1,5]

[1] Institute for Medical Informatics and Biometry (IMB), Carl Gustav Carus Faculty of Medicine, Technische Universität Dresden, Dresden, Germany
[2] Division of Stem Cells and Cancer, German Cancer Research Center (DKFZ), Heidelberg, Germany
[3] Department of Medicine, Hematology/Oncology, Goethe University Hospital Frankfurt, Frankfurt, Germany
[4] Department of Hematology, Oncology and Tumorimmunology, Charité University Medicine Berlin, Campus Virchow Klinikum, Berlin, Germany
[5] National Center for Tumor Diseases (NCT), Dresden, Germany

Contact: michael.seifert@tu-dresden.de

Contents

1 Supplementary Figures **2**

1.1 Supplementary Figure 1: Analysis of mutational burden of short- and long-lived patients and of gene expression subgroups . . . 2
1.2 Supplementary Figure 2: Extended survival analysis. . . . 3
1.3 Supplementary Figure 3: Extended analysis of short- and long-lived gene mutation profiles. 4
1.4 Supplementary Figure 4: Accumulation of *FLT3* or/and *NPM1* mutations in dependency of R882 and non-R882 *DNMT3A* mutations. . . . 5
1.5 Supplementary Figure 5: Prediction quality of the regulatory network. . . . 6
1.6 Supplementary Figure 6: Survival analysis of DNMT3A-mutant AML patients according to their FLT3 mutation type. . . . 7
1.7 Supplementary Figure 7: Additional stratification of ELN 2010 risk groups assigned to patients of the German-Austrian AML Study Group. . . . 8
1.8 Supplementary Figure 8: Additional stratification of ELN 2010 risk groups assigned to patients of the Ulm cohort. . . . 9
1.9 Supplementary Figure 9: Additional stratification of ELN 2017 risk groups. . . . 10

1 Supplementary Figures

1.1 Supplementary Figure 1: Analysis of mutational burden of short- and long-lived patients and of gene expression subgroups

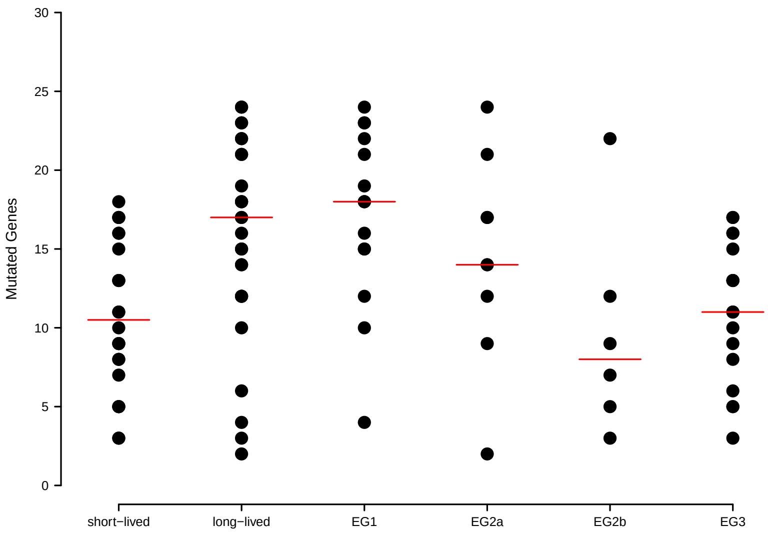

Supplementary Figure 1: Comparison of mutational burden for patients classified as short- and long-lived and for further subgrouping of patients into gene expression subgroups EG1, EG2a, EG2b, and EG3. Each black dot represents the number of mutated genes for an AML patient within the specific group. The red lines represent the median number of mutated genes within each group. The median number of mutated genes of short-lived patients is significantly smaller than the median number of mutated genes of long-lived patients (U-Test: $P < 0.004$; short-lived: 10.5; long-lived: 17). The median number of mutated genes for patients within EG2b and EG3 was significantly smaller than for patients within EG1 and EG2a (U-Test: $P < 0.002$; EG2b and EG3: 11; EG1 and EG2a: 17). Thus, there is no increased number of gene mutations for patients classified to have a poor prognosis.

1.2 Supplementary Figure 2: Extended survival analysis.

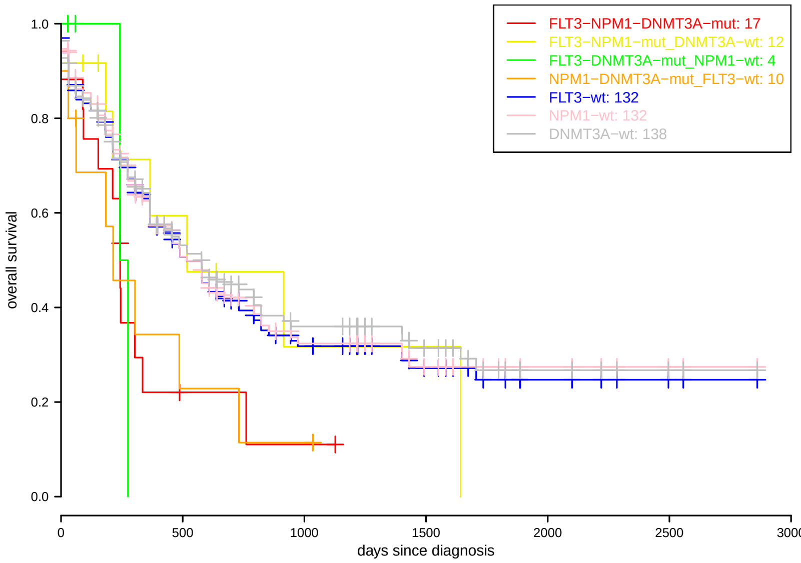

Supplementary Figure 2: Kaplan-Meier survival curves analyzing the impact of *DNMT3A*, *FLT3*, and *NPM1* co-mutations on overall survival. All AML patients with available survival information form the TCGA cohort were considered and assigned to the different groups according to their mutational status (mut: mutated, wt: not mutated) of the three genes. The numbers of patients in each subgroup are provided in the legend. As shown in Fig. 1D, the overall survival of patients with co-mutations of all three genes (red) is shorter than those of patients with *NPM1* and *FLT3* co-mutations (yellow). The survival of patients with *NPM1* and *FLT3* co-mutations (yellow) is very similar to those of patients without a *FLT3* (blue), *NPM1* (pink), or *DNMT3A* (grey) mutation for about 1600 days after diagnosis. In addition, the survival of patients with co-mutations of all three genes (red) is very similar to those of patients with *NPM1* and *DNMT3A* co-mutations (orange). Patients with *DNMT3A* and *FLT3* co-mutations show very poor survival, but this group only contains four patients, which is too few to derive a robust trend. Generally, the overall survival of patients with *DNMT3A* mutation (red, green, orange) tends to be clearly shorter than those of patients without a *DNMT3A* mutation. Thus, this analysis indicates that a *DNMT3A* mutation in combination with a *FLT3* mutation (green), a *NPM1* mutation (orange) or a co-mutation of both genes (red) contributes to shorter overall survival of affected patients of the TCGA AML cohort.

1.3 Supplementary Figure 3: Extended analysis of short- and long-lived gene mutation profiles.

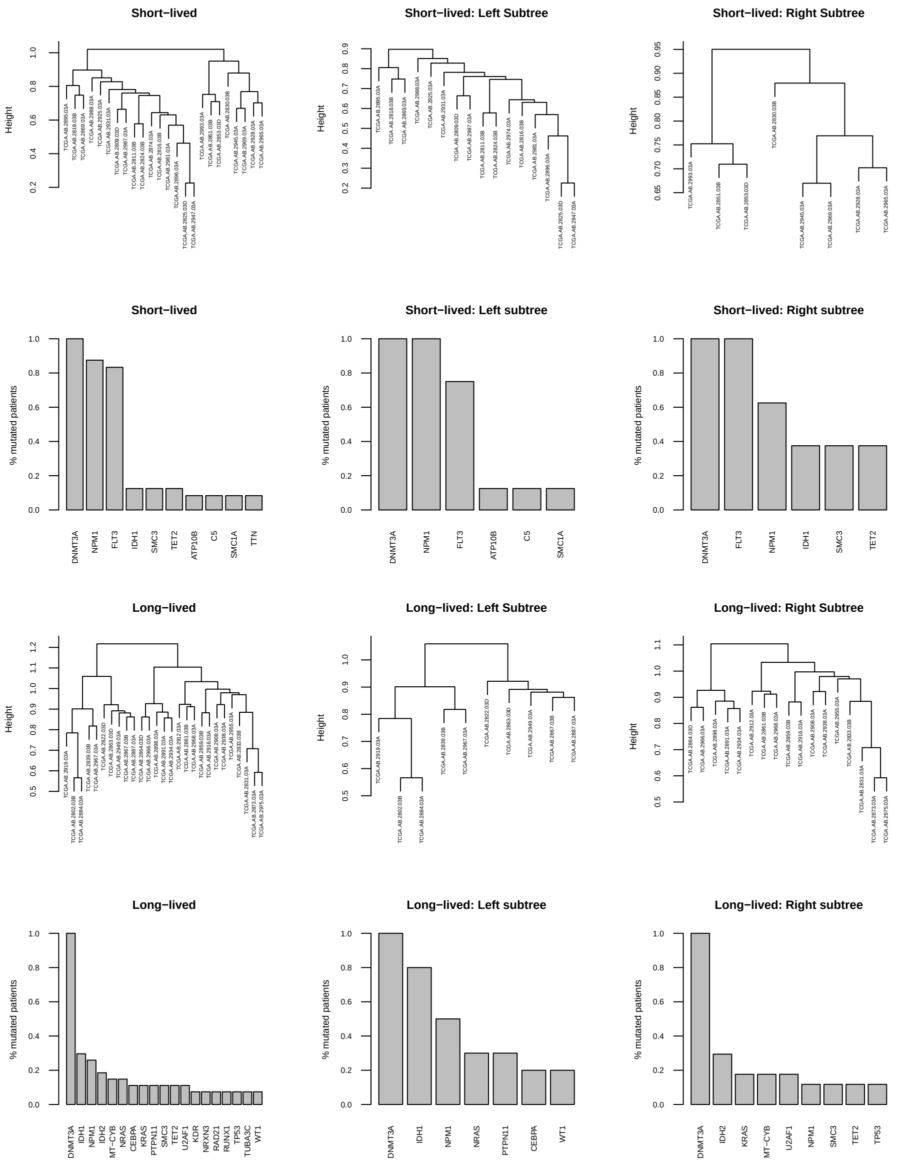

Supplementary Figure 3: Additional splitting of the dendrograms of the short- and long-lived patients into corresponding left and right subtrees (short-lived: first row, long-lived: third row). Proportions of specifically mutated genes are shown below each dendrogram (short-lived: second row, long-lived: fourth row). Only genes that were mutated in at least two patients are shown. The left and right subtree of the short-lived patients strongly differ in the number of *FLT3* and *NPM1* mutations, where within the left subtree all patients had a *DNMT3A-NPM1* co-mutation and all patients of the right subtree had a *DNMT3A-FLT3* co-mutation. The left and right subtree of the long-lived patients strongly differ in their characteristic gene mutations. A high proportion of *DNMT3A-IDH1* co-mutations is observed for patients of the left subtree, whereas *IDH1* mutations were not observed in the right subtree, which includes several patients with *DNMT3A-IDH2* co-mutations instead. Also the proportion of *NPM1* mutations differs between these two subtrees.

1.4 Supplementary Figure 4: Accumulation of *FLT3* or/and *NPM1* mutations in dependency of R882 and non-R882 *DNMT3A* mutations.

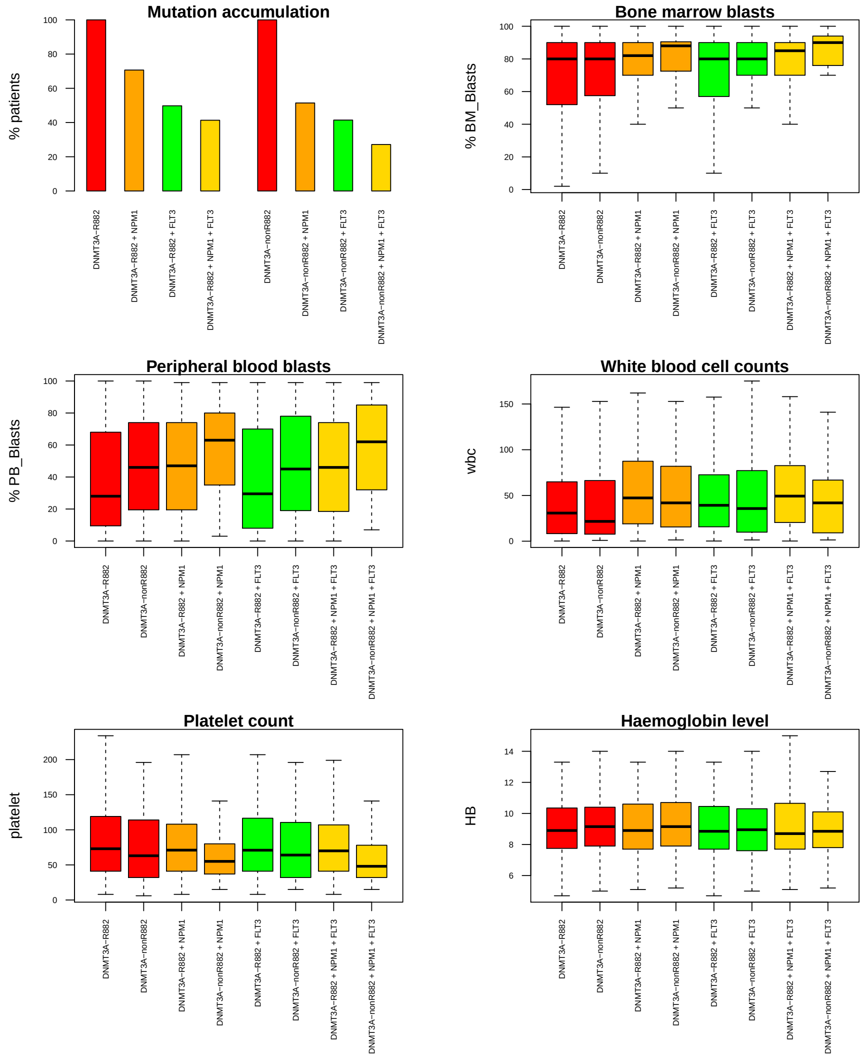

Supplementary Figure 4: We identified an enrichment of *DNMT3A*-R882 mutations in the short-lived group compared to the long-lived group in our analysis of *DNMT3A*-mutant AML patients from TCGA. All short-lived patients also had *NPM1* and/or *FLT3* mutations that were only observed for few patients of our long-lived group. This raised the question if *NPM1*, *FLT3* or a co-mutation of both genes is observed to a greater proportion in AML patients with *DNMT3A*-R882 mutations compared to *DNMT3A*-non-R882 mutations? We therefore analyzed gene mutation data of a very large cohort of 1,540 AML patients form the German-Austrian AML Study Group published by *Gerstung, M. et al. (2017)*, Nat Genet, 49, 332-340 (https://github.com/gerstung-lab/AML-multistage/blob/master/data/AMLSG_Genetic.txt). This data set contained 362 patients with *DNMT3A* mutations of which 222 patients had a *DNMT3A*-R882 mutation, 137 had another *DNMT3A* mutation (nonR882), and 3 patients had both types of *DNMT3A* mutations. We then determined for the group of *DNMT3A*-R882-mutant and the group of *DNMT3A*-nonR882-mutant patients the proportion of patients that also had a *NPM1*, a *FLT3*, or a co-mutation of both genes (subfigure: Mutation accumulation) and further tested if any of these mutations differed in their proportion of affected patients between both groups using Fisher's exact test. We found a significant enrichment of *DNMT3A*-R882 and *NPM1* co-mutations and a significant enrichment of concurrent *DNMT3A*-R882, *NPM1*, *FLT3* mutations compared to the corresponding groups of patients that had another *DNMT3A* mutation (DNMT3A-R882

1.5 Supplementary Figure 5: Prediction quality of the regulatory network.

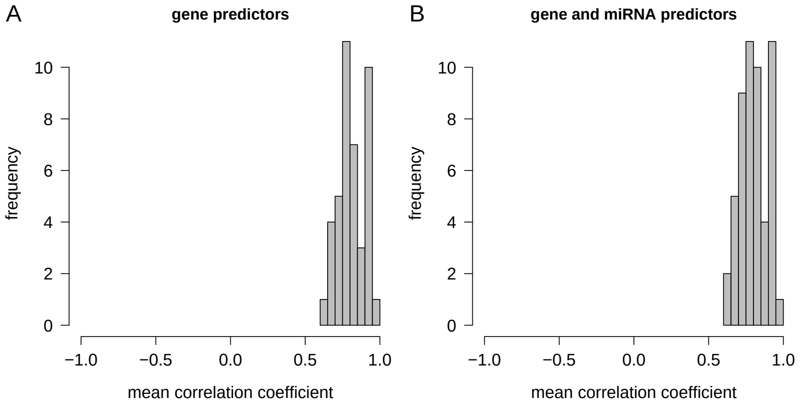

Supplementary Figure 5: Prediction accuracy of gene expression levels by regulatory networks. Correlations between network-based predicted and experimentally measured gene expression levels were determined for the test samples that were not included in network inference. Significant shifts of the mean correlations into the positive range were observed for networks learned based on gene expression (A) and gene expression and microRNA expression data (B) (Wilcoxon signed rank test: $P < 0.0001$).

1.6 Supplementary Figure 6: Survival analysis of DNMT3A-mutant AML patients according to their FLT3 mutation type.

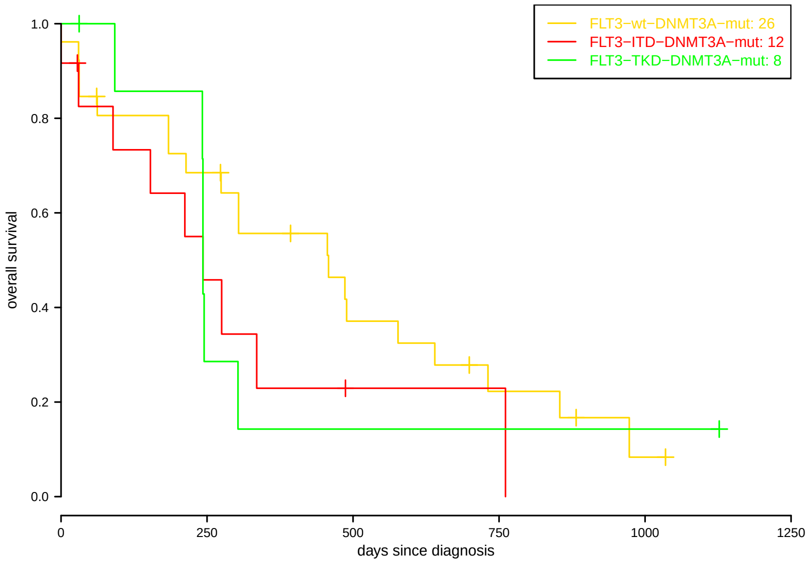

Supplementary Figure 6: Kaplan-Meier survival curves distinguishing *DNMT3A*-mutated AML patients from TCGA according to their *FLT3* mutation status. *DNMT3A*-mutant patients with FLT3-ITD (red) and FLT-TKD (green) show similar survival curves that do not differ significantly. Both curves do also not differ significantly from *DNMT3A*-mutant patients without FLT3 mutations (orange). Numbers of patients with available survival information are given in the legend for each of the three groups. An additional stratification based on the *FLT3* mutation type does not further improve our classification of *DNMT3A*-mutated AML patients from TCGA.

1.7 Supplementary Figure 7: Additional stratification of ELN 2010 risk groups assigned to patients of the German-Austrian AML Study Group.

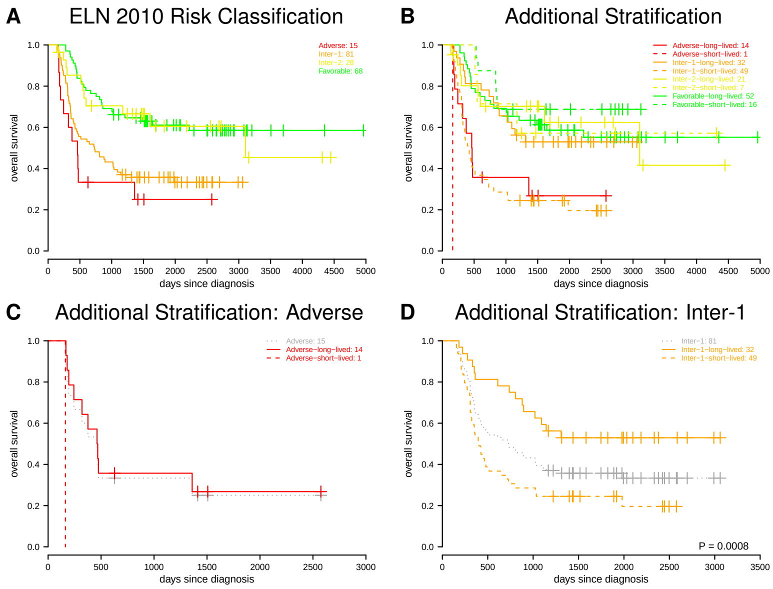

Supplementary Figure 7: Impact of our short- and long-lived classification on the ELN risk classification of independent validation patients from the German-Austrian AML Study Group. (A) Basic Kaplan-Meier curves according to publicly available ELN risk classification (adverse, inter-1, inter-2, favorable) of independent validation patients from the German-Austrian AML Study Group. (B) Additional stratification of the patients in the ELN risk categories based on our classification of patients as short- and long-lived. Patients were assigned to short- or long-lived based on their gene mutations. (C and D) Separate visualizations of the additional stratification of adverse (C) and inter-1 patients (D) that showed the strongest benefit from our additional stratification. The basic Kaplan-Meier curve of the original ELN risk category is shown as grey dotted curve. Patients of this risk category that were classified as long-lived are shown by a solid Kaplan-Meier curve and patients classified as short-lived are shown by a dashed Kaplan-Meier curve. Too few patients were in the adverse-short-lived subgroup to support the observed difference between both curves by a log-rank test (C), but the benefit of our additional classification is clearly demonstrated for the inter-1 risk group (D), where the inter-1-long-lived group differs significantly in survival from the inter-1-short-lived subgroup (log-rank test: $P = 0.0008$).

1.8 Supplementary Figure 8: Additional stratification of ELN 2010 risk groups assigned to patients of the Ulm cohort.

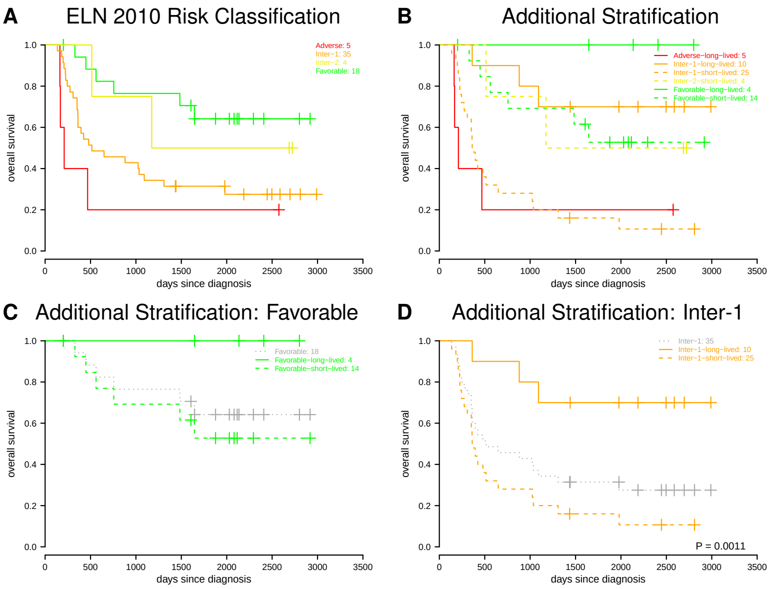

Supplementary Figure 8: Impact of our short- and long-lived classification on the ELN risk classification of independent validation patients from the Ulm cohort. (A) Basic Kaplan-Meier curves according to publicly available ELN risk classification (adverse, inter-1, inter-2, favorable) of independent validation patients from the Ulm cohort. (B) Additional stratification of the patients in the ELN risk categories based on our classification of patients as short- and long-lived. Patients were assigned to short- or long-lived based on their gene expression profiles. (C and D) Separate visualizations of the additional stratification of favorable (C) and inter-1 patients (D). The basic Kaplan-Meier curve of the original ELN risk category is shown as grey dotted curve. Patients of this risk category that were classified as long-lived are shown by a solid Kaplan-Meier curve and patients classified as short-lived are shown by a dashed Kaplan-Meier curve. Too few patients were in the favorable-long-lived subgroup to support the observed difference between both curves by a log-rank test (C), but the benefit of our additional classification is again clearly demonstrated for the inter-1 risk group (D), where the inter-1-long-lived group differs significantly in survival from the inter-1-short-lived subgroup (log-rank test: $P = 0.0011$).

1.9 Supplementary Figure 9: Additional stratification of ELN 2017 risk groups.

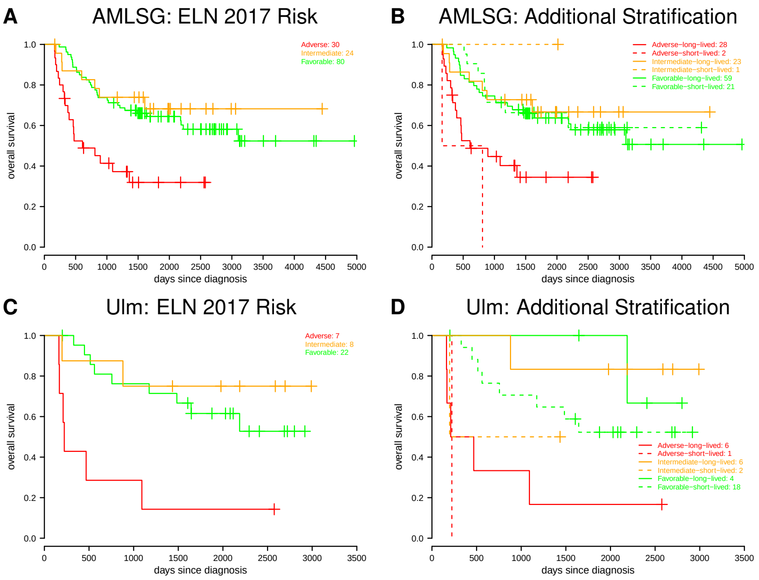

Supplementary Figure 9: Impact of our short- and long-lived classification on the revised ELN 2017 risk classification. Only a subset of the validation patients could be included, because a reclassification according to the ELN 2017 system requires data on *FLT3*-ITD-to-wild-type allelic ratio for affected patients. These allelic ratios were not publicly available. Therefore, we could only consider patients that were reliably reclassified by Herold et al. (2020) [77] excluding almost all *DNMT3A*-mutant AML validation patients with a known *FLT3*-ITD mutation. (A) Basic Kaplan-Meier curves for the ELN 2017 risk classification (adverse, intermediate, favorable) for the subset of independent validation patients from the German-Austrian AML Study Group that could be reclassified (134 of 208). Not as expected, the intermediate risk group tends to perform better than favorable risk group in this small validation cohort. (B) Additional stratification of the patients in the ELN 2017 risk categories based on our classification of patients as short- and long-lived. Patients were assigned to short- or long-lived based on their gene mutations. Similar to Supplementary Figure 7B, our additional stratification has no positive or negative impact on the favorable risk group. Too few patients were part of the other risk groups for a statistical analysis of potential trends. (C) Basic Kaplan-Meier curves for the ELN 2017 risk classification (adverse, intermediate, favorable) for the subset of independent validation patients from the Ulm cohort that could be reclassified (37 of 63). Again, the intermediate risk group tends to perform better than favorable risk group. (D) Additional stratification of the patients in the ELN 2017 risk categories based on our classification of patients as short- and long-lived. Patients were assigned to short- or long-lived based on their gene expression profiles. Similar to Supplementary Figure 8B, our additional stratification may have the potential to refine the classification of the favorable and intermediate risk group. Too few patients were in the risk groups for a statistical analysis of these trends.
